# Unlabeled but Not Unseen: Cytotoxicity Classification of Re(I) Tricarbonyl Complexes via K‑Means Clustering

**DOI:** 10.1021/acsomega.5c13156

**Published:** 2026-06-11

**Authors:** Miroslava Nedyalkova, Gozde Demirci, Youri Cortat, Lilai Abraha, Aurelien Crochet, Marco Lattuada, Fabio Zobi

**Affiliations:** † Department of Chemistry, 27211 University of Fribourg, Chemin Du Musée 9, 1700 Fribourg, Switzerland; ‡ Swiss National Center for Competence in Research (NCCR) Bio-Inspired Materials, University of Fribourg, 1700 Fribourg, Switzerland; § Faculty of Chemistry and Pharmacy, Sofia University “St. Kliment Ohridski”, 1 James Bourchier Blvd., 1164 Sofia, Bulgaria; ∥ Research and Development and Innovation Consortium (Sofia Tech Park), 111 Tsarigradsko Shosse Blvd., 1784 Sofia, Bulgaria

## Abstract

The prediction of cytotoxicity for metal-based drug candidates remains a significant challenge due to the structural diversity and multifactorial mechanisms of action inherent to transition metal complexes. Here, we present an unsupervised machine learning approach employing K-means clustering to cluster the cytotoxicity of 225 rhenium­(I) tricarbonyl complexes based solely on molecular descriptors. After comprehensive descriptor calculation and reduction, principal component analysis was used to assess chemical space coverage and identify key variables. K-means clustering, applied without prior toxicity labels, successfully partitioned the data set into cytotoxic and noncytotoxic clusters, achieving high concordance with known biological activity and accurately clustering control compounds. Analysis of misclassified cases provided further insight into structural motifs associated with ambiguous toxicity profiles. This work demonstrates that K-means clustering, when integrated with robust descriptor selection and PCA, offers a transparent, efficient, and interpretable framework for early stage toxicity assessment in metal-based drug discovery, particularly when labeled data are limited.

## Graphical abstract

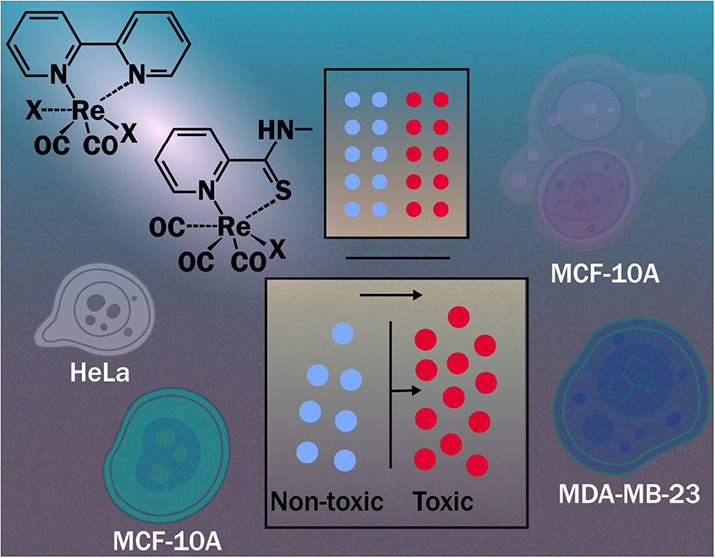

## Introduction

In recent years, machine learning (ML) approaches have gained substantial traction for predicting the toxicity of chemical compounds based on their molecular structures, and are occasionally supplemented by additional data sources.^1^ These methods have shown particular promise in drug discovery, where there is a growing need to reduce costs and improve efficiency. Despite continuous innovation in applying ML to biological systems—ranging from drug–target interaction modeling to adverse effect prediction—practical implementation remains limited. ML models, including Quantitative Structure–Activity Relationship (QSAR) and Quantitative Structure–Property Relationship (QSPR) models, can be trained on empirical assay data to predict key physicochemical or biological properties directly from molecular descriptors.^2^ Furthermore, rapid in silico screening pipelines enable for high-throughput assessment of compound libraries, significantly reducing the time and resources needed for experimental validation.

A recent comprehensive review^3^ highlights the growing importance of ML in toxicity prediction, emphasizing various toxic end points such as acute oral toxicity, hepatotoxicity, cardiotoxicity, mutagenicity, and the Tox21 data set end points. The study discusses the diversity of ML models applied, the impact of molecular representations, algorithms, and evaluation metrics, and the challenges in comparing performance across different toxicological end points. Such insights underscore the complexity of toxicity prediction and the ongoing evolution of computational strategies in this field.

The implementation of these methods for the prediction and rationalization of cytotoxicity profiles is a critical objective in the early development of metal-based drug candidates (or metallodrugs), as these compounds often exhibit diverse mechanisms of action and interact with biological targets in ways that differ fundamentally from traditional organic-based drugs. Unlike classical small molecules, metal complexes can engage in a broader array of biological interactions due to their properties such as redox activity, variable coordination numbers, ligand exchange dynamics, and three-dimensional geometries.^4–6^ This is particularly relevant for transition metal complexes, which have gained increasing attention for their therapeutic potential in antimicrobial, anti-inflammatory, and anticancer applications.^7,8^ However, their multifactorial modes of action and structural diversity complicate the extraction and interpretation of consistent structure–activity trends, especially when descriptor representations and experimental conditions vary across studies.^9^ As a result, computational approaches for toxicity prediction must account for both structural and mechanistic heterogeneity. In this context, it is important to note that the present study is based on a classical descriptor-based QSAR framework.^10,11^

The novelty of this work lies not in algorithmic advancement, but in the application of unsupervised K-means clustering to systematically explore structure–cytotoxicity trends in a chemically complex class of rhenium­(I) tricarbonyl complexes, using molecular descriptors without incorporating cytotoxicity data during cluster formation.

The most commonly employed models for toxicity prediction include k-nearest neighbors (kNN),^12^ support vector machines (SVM),^13^ random forests (RF),^14^ and algorithms based on artificial neural networks (NN), including those utilizing deep learning (DL) techniques.^15–17^ Among these, DL-based approaches have become particularly prominent, leveraging multiple layers of neural networks to capture complex patterns in data. Deep learning encompasses a wide array of architectures, with the most frequently used being multilayer perceptrons (MLP).^18^

The application of ML to transition metal complexes has progressed relatively slowly, primarily due to the limited availability of well-curated data sets. Nonetheless, Kulik’s group has made significant contributions to advancing ML-driven strategies for exploring large chemical spaces and optimizing transition metal complexes.^19–21^ In contrast to recent large-scale supervised studies such as that of Krasnov et al.^22^ which leverage extensive multi–cell line data sets to build predictive models, the present data set reflects the typical constraints of metallodrug research: heterogeneous data, limited overlap in biological assays, and incomplete labeling. Under these conditions, unsupervised learning provides a complementary strategy focused on identifying robust structure–activity trends rather than optimizing predictive accuracy. Balcells et al. introduced a novel representation tailored for deep graph learning applied to transition metal complexes.^23^ In the realm of new antimicrobial discovery Frei et al. have applied ML to 288 ruthenium arene Schiff-base complexes, successfully predicting 54 new metalloantibiotic candidates.^24^ Considering the existing ML algorithms, a key challenge in applying these learning models in predictive toxicology, lies in interpreting the predictions they generate, especially when deep learning models are involved. K-Means clustering offers a practical alternative to traditional supervised learning approaches for toxicity particioning, particularly when working with limited or incomplete labeled data. For this reason, using not only deep learning (DL) models is the best choice. DL models have a higher predictive accuracy in toxicity groups, but they have limitations such as high data requirements, low interpretability, and significant computational demands in, e.g., defining a set of constraints. In contrast, K-Means clustering offers a simpler and more interpretable alternative, particularly valuable in scenarios with limited labeled data, such as early stage screening of metal-based compounds. K-Means requires no prior knowledge of toxicity labels and can still reveal meaningful patterns by grouping compounds with similar structural or physicochemical characteristics. This unsupervised approach facilitates rapid exploratory analysis, enabling researchers to hypothesize toxic or nontoxic behavior based on cluster composition.

While it does not rely on predefined labels during training, K-Means can still reveal meaningful structure–toxicity relationships, making it especially useful in exploratory phases of compound screening or in underrepresented chemical spaces such as metal-based complexes. Although its performance may not match that of supervised models in terms of predictive accuracy, its simplicity, speed, and interpretability make it a valuable tool for early stage analysis and hypothesis generation in toxicity assessment pipelines.

In this paper our focus is on rhenium­(I) tricarbonyl complexes, which have emerged as promising candidates in both anticancer^25–29^ and antimicrobial therapy^30–34^ due to their low in vivo toxicity, low effective concentrations, structural tunability and chemical stability. These complexes can be further functionalized to target specific cellular compartments or to enhance selectivity toward cancer cells or microbial membranes.^35^ Previous studies have demonstrated the cytotoxic efficacy of various rhenium­(I) tricarbonyl complexes against cancer cell lines.^26^ For instance, phenanthroline-based derivatives have shown significant activity against triple-negative breast cancer cells, with IC_50_ values below 5 μM. Additionally, certain aminoquinoline-based complexes have exhibited potent anticancer properties, highlighting the versatility of the rhenium­(I) tricarbonyl core in medicinal chemistry.^36^ To address the possibility of early cytotoxicity assessment of such complexes, we introduce the use of K-Means clustering as a strategic, unsupervised learning tool that offers a transparent, efficient, and label-free framework for discriminating toxic from noncytotoxic compounds based solely on molecular descriptors. The K-means clustering is label-free during model construction, and cytotoxicity labels are applied only a posteriori for interpretation.

## Materials and Methods

### Data Set Structure

The literature was searched for a large representative number of complexes of the fac-[Re­(CO)_3_]^+^ core evaluated in vitro for their anticancer cytocytotoxic properties (IC_50_ values). Data was extracted from different sources, but mainly from available reviews on the subject.^26–28^ The work of Wilder et al. also provided several data points for our analysis.^37^ Dicarbonyl complexes of the *cis*-[Re­(CO)_2_]^+^ core was also added to the search.^38^ Both mononuclear (ca. 95% of final total) and dinuclear species, mainly with mono- and bidentate ligands, were included in the pool. This initial selection of candidate molecules was further analyzed in terms of the cancer cell lines used in their in vitro evaluation. Cytotoxicity of small molecules and metal complexes is known to depend on cellular context, including cell line–specific uptake, metabolism, and stress-response pathways. In the present work, this variability is not treated as a variable to be resolved at the level of individual cell lines, but rather as background experimental noise intrinsic to literature-derived data sets. Accordingly, the analysis focuses on identifying structure-driven cytotoxicity propensities that are robust across heterogeneous biological assays, rather than on cell line–specific potency. Cytotoxicity data were collected from the literature and originate from multiple studies employing different cancer cell lines (refences indicated in the SI data set). Not all Re­(I) tricarbonyl complexes in the data set were evaluated against the same cell line, reflecting the heterogeneity of available experimental data. When multiple IC_50_ values were reported for a given compound across different studies or cell lines, a single representative value corresponding to the most frequently reported experimental conditions was retained to ensure data set consistency.These are the HeLa, MCF7, MCF10A and MDA-MB-231cancer cell lines.

A data set of 225 Re­(I) tricarbonyl complexes was assembled, with IC_50_ values and K-Means clustering was used to define toxicity groups based on descriptor space separation. This approach allowed us to identify natural groupings in the chemical space that could correspond to toxicity clusters, enabling toxicity assessment without relying on predefined labels from inconsistent or incomplete experimental data. The complexes were described by one-dimensional (1D) and two-dimensional­(2D) molecular descriptors generated by the AlvaDesc software.^39^

Prior to dimensionality reduction and clustering, descriptors exhibiting near-zero variance across the data set were removed. Near-zero variance was defined as descriptors with a variance below 1 × 10^–6^ after standardization. To reduce multicollinearity, pairwise Pearson correlation coefficients were computed, and for any pair of descriptors with an absolute correlation coefficient |*r*| ≥ 0.95, one descriptor was removed.

The ESI contains the complete data set used in this study, including SMILES representations, and IC_50_ values assigned to specific cell lines. Additionally, all calculated physicochemical descriptors (e.g., molecular weight, *M* log *P*, and LOGPcons) are provided, together with extended data visualizations and supplementary analyses supporting the main manuscript. (presented in the SI and uploaded at the following link: https://zenodo.org/records/19698608) after excluding those with missing values, constant or near-constant values, and high intercorrelation.

#### Multivariate Analysis and Clustering of the Descriptor Space

To analyze and interpret the chemical space, Principal Component Analysis was applied as an unsupervised dimensionality reduction technique, enabling the identification of dominant patterns by projecting the data onto new orthogonal axes (principal components).^40^ As a linear method, PCA captures only linear relationships among descriptors and was therefore selected primarily for its interpretability, rather than for optimizing cluster separation.

To determine which descriptors were most influential in shaping the chemical space, the contribution of each descriptor to the first two principal components (PC1 and PC2) was quantified using their loading scores. A total importance score was calculated for each descriptor as the sum of the absolute values of its loadings on PC1 and PC2, serving as a measure of overall influence on the chemical space distribution.

The distribution of all compounds along these two principal components is shown in Table S12 in the SI, providing a direct visualization of the reduced chemical space. As well the obtanaed data for the PCA revealed that PC1 and PC2 accounted for 36.8% and 10.5% of the total variance in the standardized descriptor space, respectively, capturing 47.4% of the overall variance. The relatively high variance captured by PC1 reflects substantial descriptor redundancy arising from correlated physicochemical and topological features, whereas PC2 captures orthogonal structural variation, consistent with the high-dimensional nature of molecular descriptor space.

A complete ranking of all molecular descriptors based on their total importance (|PC1 loading| + |PC2 loading|) is provided in separate file in the SI. The importance of individual molecular descriptors was quantified using their loading values on the first two principal components. For each descriptor, a total importance score was defined as the sum of the absolute values of its loadings on PC1 and PC2 (|loading_PC1| + |loading_PC2|). This metric captures the overall contribution of a descriptor to the two-dimensional chemical space representation, independent of loading sign.

Descriptors with high total importance values contribute strongly to at least one of the two principal components and therefore play a dominant role in shaping the variance structure of the chemical space. In practice, high-ranking descriptors typically correspond to physicochemical, topological, or surface-related properties that are strongly correlated with multiple features in the descriptor set, thereby driving large-scale separation along PC1 or orthogonal variation along PC2. The Top 20 descriptors ranked by this metric represent those variables with the greatest influence on the PC1–PC2 projection and thus provide insight into the dominant structural and physicochemical factors underlying the observed organization of the molecular data set.

For further investigation of the chemical space structure, we applied K-means clustering, an unsupervised learning algorithm that partitions data into clusters based on feature similarity. The clustering was performed without using toxicity labels for the metal complexes in the data set. Subsequently, we compared the clustering results to the known toxicity groups (cytotoxic vs noncytotoxic) to evaluate whether the natural groupings in the descriptor space correspond to defined biological activities or responses. This approach revealed whether the features driving unsupervised clustering were also relevant to toxicity classification. In the context of cytotoxicity-based molecular partitioning of rhenium tricarbonyl complexes, such analysis provides insights into structure–activity relationships and highlights the electronic and structural properties that may govern cytotoxicity. For the purposes of classification limits, compounds with IC_50_ values below 10 μM were considered cytotoxic, following an accepted thresholds in drug discovery and toxicology.^41,42^

#### K-Means Clustering Analysis

K-means clustering was applied to identify groups of Re­(I) tricarbonyl complexes with similar molecular descriptor profiles in a multidimensional feature space by minimizing within-cluster variance in standardized descriptor space. K-means clustering is an unsupervised partitioning algorithm that assigns data points to a predefined number of clusters (*k*) by minimizing the within-cluster sum of squared distances between each point and its corresponding cluster centroid. The algorithm proceeds iteratively by initializing centroids (typically using random or heuristic-based initialization), assigning each data point to the nearest centroid according to a chosen distance metric, and subsequently updating centroid positions based on the mean of the assigned points. This process is repeated until convergence, defined as no further change in cluster assignments or centroid positions, resulting in clusters characterized by high internal similarity and reduced variance within each group.^43^ Depending on the choice of distance metric, the way similarity is assessed can vary. In this study, the Euclidean distance metric was selected for use with the K-means algorithm.^44,45^ Statistica software (version 9) was used for the exploratory data analysis.

## Experimental Section

The data set of Re­(I) tricarbonyl complexes comprises 14 complexes which were not previously tested as anticancer compounds. Of these, 7 are new molecules.

We have measured the cytotoxicity of the 14 complexes in HeLa cell. The compounds of general formula [Re­(CO)_3_(NN)­L]^n^ (where NN = diimine ligand, L = monodentate ligand and *n* is charge, 0 or +1, depending on L) are of the following ligand combination: NN = 2,2′-bipyridine (bpy, 1); 1,10-phenanthroline (phen, 2); 3,4,7,8-tetramethyl-1,10-phenanthroline (tmetphen, 3); 2,9-dimethyl-1,10-phenanthroline (dimetphen, 4); bathophenanthroline (bathophen, 5); bathocuproine (bathocup, 6); L = Br^–^, (a); 1-methyl-1H-imidazole (MeIm, b); benzoate (Ben, c). Thus, e.g., [Re­(CO)_3_(bpy)­Ben] is coded “1c” or [Re­(CO)_3_(phen)­MeIm]^+^ is coded “2b” etc. Note that all cationic complexes are CF_3_SO_3_^–^ salts. Below we detail the preparation of the new compounds, in addition the X-ray structure of 7 complexes (all previously unknown) is given in Figure 1.

**Figure 1:**
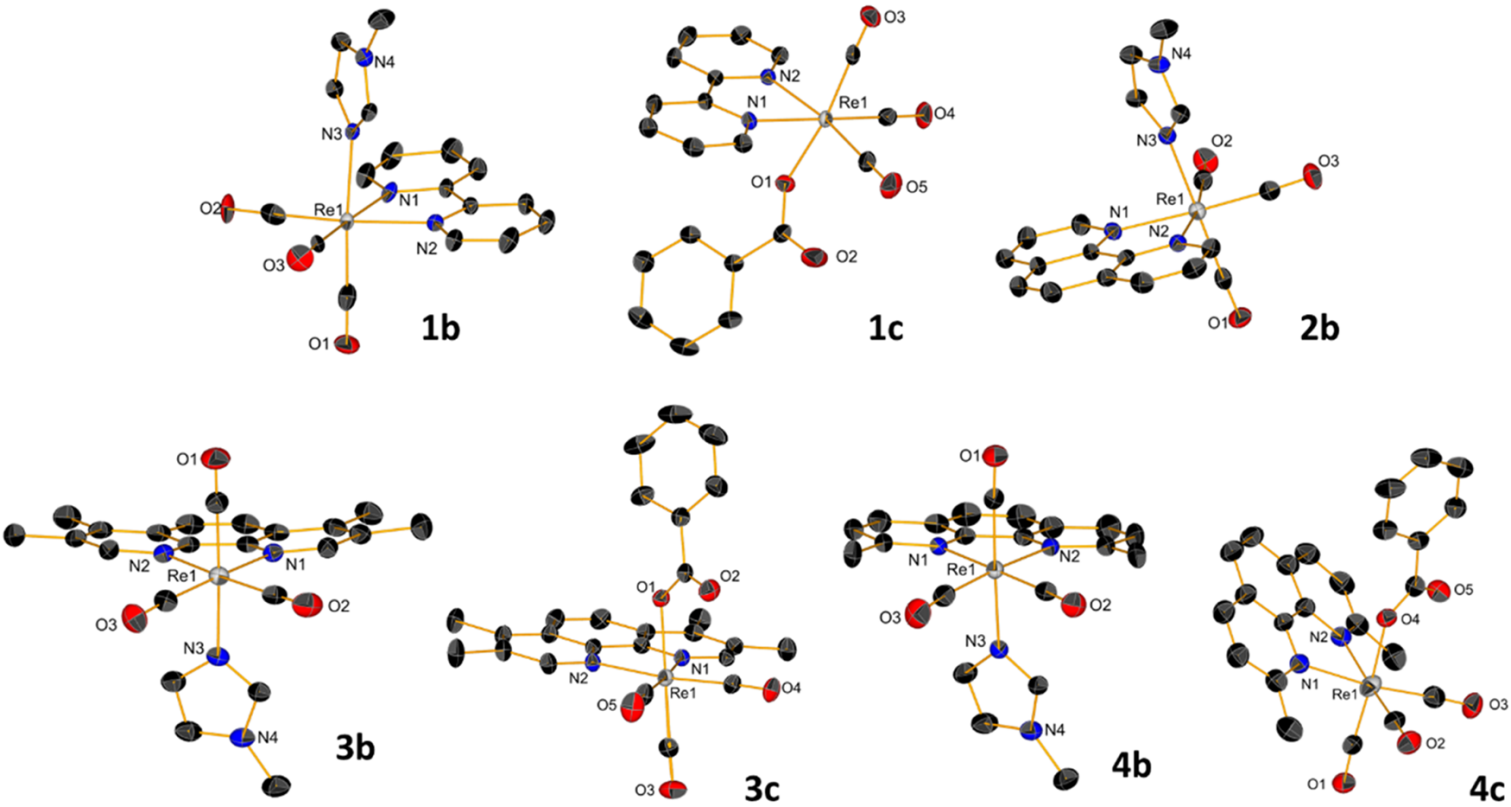
Single-crystal X-ray diffraction structures of selected molecules. Thermal ellipsoids are at 30% probability. Hydrogen atoms and counterions (1b, 2b, 3b and 4b) are omitted for clarity.

Synthesis of rhenium complexes. All complexes were synthesized according to previous reported procedures.^30,46–49^ In general, 1-Methylimidazole complexes were synthesized with AgCF_3_SO_3_ salt in MeOH at 55 °C for overnight. Benzoate complexes were synthesized according to literature.^50^ See SI for details and characterization of molecules.

## Results and Discussion

### Chemical Space and Physicochemical Distribution of the Data Set

To ensure full transparency and provide a comprehensive overview of the chemical space explored in this study, we analyzed the physicochemical distribution of the data set, including molecular weight (MW) and lipophilicity descriptors (*M* log *P* and LOGPcons).

Scatter plots of IC_50_ values against MW and lipophilicity descriptors (Figure 2A–C) reveal the absence of trivial correlations, indicating that biological activity is not governed by a single physicochemical parameter. This suggests a complex interplay between structural features and activity. The distribution plots (Figure 2D–F) demonstrate a broad coverage of chemical space, confirming that the data set spans diverse molecular scaffolds and physicochemical regimes relevant to drug discovery. Overall, these analyses demonstrate substantial chemical diversity and support the absence of simple structure–activity relationships, motivating the use of multivariate or data-driven approaches to rationalize biological activity.

**Figure 2:**
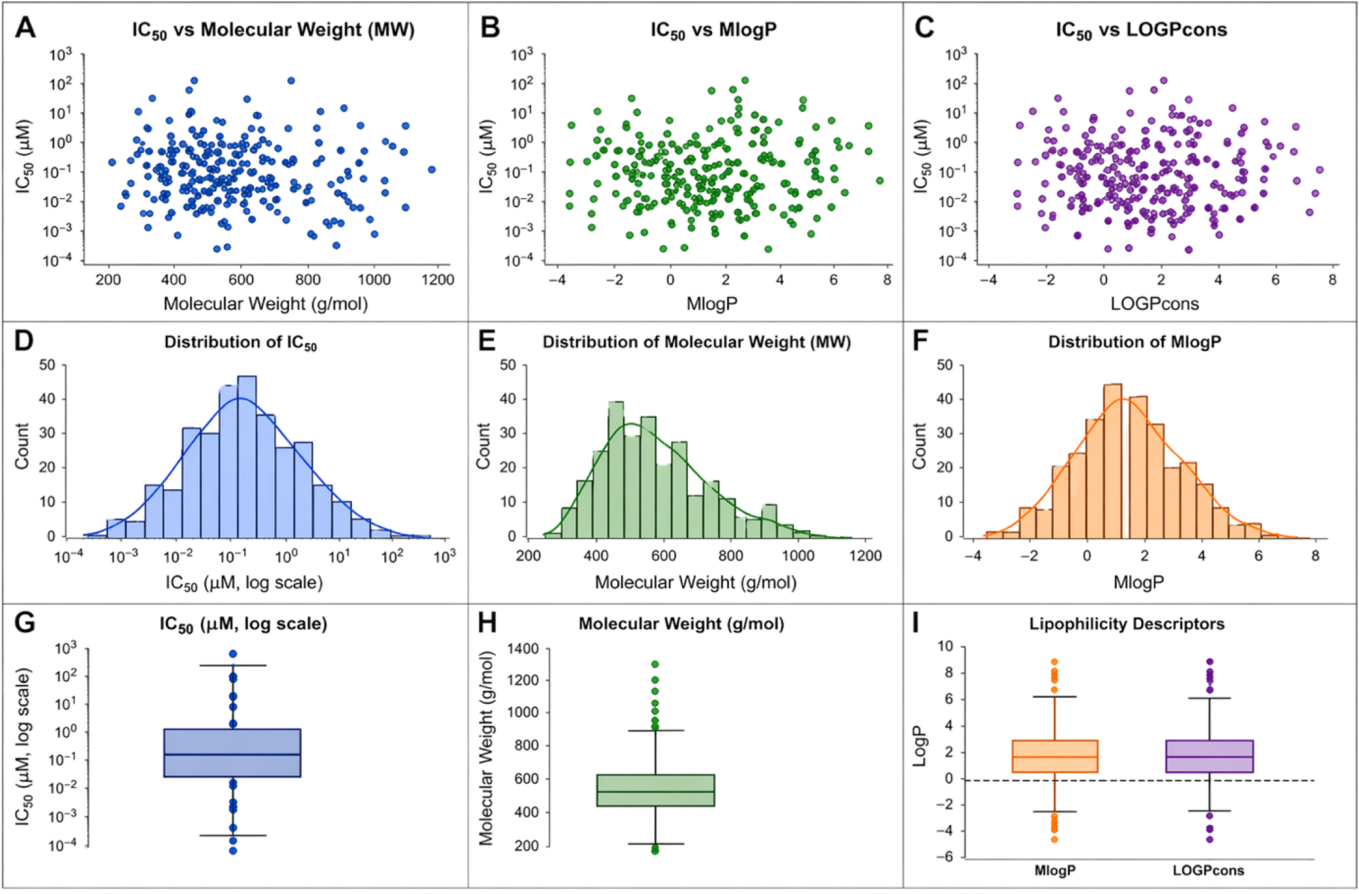
Chemical space and physicochemical characterization of the data set. (A–C) Scatter plots showing the relationship between IC_50_ values and key physicochemical descriptors: molecular weight (MW), *M* log *P*, and consensus Log *P* (LOGPcons). The absence of clear linear trends indicates that biological activity is not governed by a single descriptor. (D–F) Distribution histograms illustrating the spread of IC_50_ values (log scale), molecular weight, and lipophilicity (*M* log *P*), highlighting the broad and heterogeneous nature of the data set. (G–H) Box plots summarizing the distribution, central tendency, and variability of IC_50_ and molecular weight, respectively, with visible outliers indicating extreme activity or size regimes. (I) Comparative box plots of lipophilicity descriptors (*M* log *P* and LOGPcons), showing their distribution and consistency across the data set.

### Partitioning: Identification of Hidden Spatial Patterns between the Metal Complexes

To identify underlying structure in the descriptor space without imposing prior assumptions on class labels, K-means clustering was selected as a simple and interpretable unsupervised method for partitioning the data set. K-means clustering was applied to a data set of rhenium­(I) tricarbonyl complexes (*n* ≈ 225) using a comprehensive set of molecular descriptors (available at https://zenodo.org/records/19698608). A post hoc comparison with literature IC_50_ values reveals a clear but incomplete trend: Cluster 0 is enriched in noncytotoxic compounds (85%), whereas Cluster 1 is enriched in cytotoxic compounds (77.5%). This indicates that the clustering captures meaningful chemical information, while also highlighting that it does not produce fully separated biological classes.

To better understand the global organization of the data set, as we already mention the PCA was first applied to reduce the dimensionality of the descriptor space. The diffuse distribution suggests that the chemical space is continuous, with gradual transitions between molecular profiles rather than discrete partitions. Because PCA is inherently linear, Kernel PCA using an RBF kernel was additionally employed to probe potential nonlinear relationships. However, the same overall pattern was revealed even when nonlinear structure is considered, the data do not resolve into well-defined clusters.

To ensure that these observations are not artifacts of projection, K-means clustering was performed directly in the original high-dimensional descriptor space, and the resulting cluster assignments were projected onto both PCA and Kernel PCA representations for visualization. The clusters remain substantially overlapping in all representations, indicating only weak structural organization. This is quantitatively supported by silhouette analysis, which yields a mean value of ∼0.22. What we see as a result based on the silhoetets, distinct and well-separated groups are not defined, the compounds occupy overlapping regions of chemical space, meaning that many molecules share similar descriptor profiles despite belonging to different clusters. This indicates that a significant number of compounds lie in transitional regions between thde clusters groups. In other words, we can say that this behavior is chemically meaningful. The descriptors used—encompassing physicochemical, topological, and surface-related properties—capture general aspects of molecular structure but do not fully encode more subtle features such as electronic effects, coordination environment, or specific interaction mechanisms. As a result, compounds with similar descriptor profiles can still exhibit different cytotoxic responses.

The IC_50_ distributions provide an important complementary perspective. When visualized independently using violin plots, cytotoxic and noncytotoxic compounds appear partially separated. However, this apparent distinction arises from the imposed classification threshold rather than from an intrinsic separation in descriptor space. When these compounds are mapped back onto the multivariate descriptor landscape, they occupy overlapping regions, reinforcing the idea that activity is not governed by a single dominant property or clearly defined boundary.

Taken together, these results point to a consistent and coherent interpretation: the data set represents a continuous and heterogeneous chemical space in which biological activity varies smoothly rather than discretely. Within this context, K-means clustering remains useful—not as a strict classifier, but as a tool for identifying regions of enrichment and underlying trends. The findings also highlight a broader limitation of descriptor-based models: while they effectively capture global structural variation, they only partially explain biological activity, which likely depends on more nuanced, context-dependent molecular features (Figure 3).

**Figure 3:**
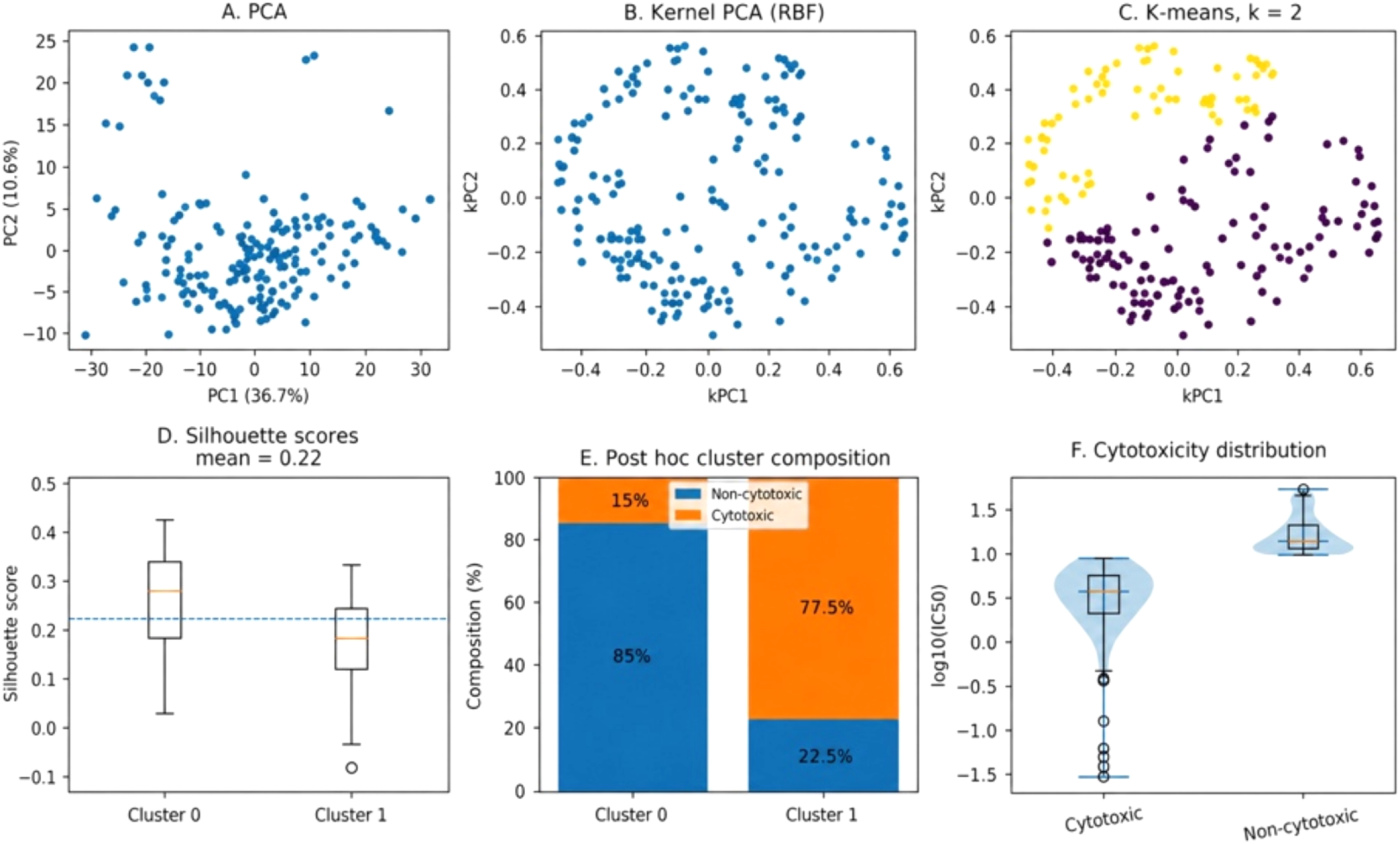
Analysis of chemical space, clustering, and cytotoxicity. (A) PCA projection of the full standardized descriptor space showing a diffuse distribution without clear separation. (B) RBF representation capturing potential nonlinear relationships, confirming the absence of distinct clusters. (C) K-means clustering (*k* = 2) performed in the original descriptor space and projected onto the Kernel PCA representation, highlighting partial grouping with substantial overlap. (D) Distribution of silhouette scores per cluster. (F) Violin plots of log-transformed IC_50_ values illustrating substantial overlap between cytotoxic and noncytotoxic compounds.

The main structural motifs observed across the clustered complexes were predominantly represented by the compound motif types labeled as A–F as presented in Figure 4. We see that we have variation from small, or compact structures - such as the *N*-methylpyridine-2-carbothioamide species of Lyczko et al.^51^ (type A) or the dicarbonyl complexes of Schindler et al.^37^ (type B) - to complexes bearing long aliphatic chains on the monodentate ligand, such as molecules of types C-E (C D E).^52–55^ Additionally, compounds bearing tridentate ligands of the bis­(pyridin- and/or quinolin-2-ylmethyl)­amine (types E and F) were also included.^56,57^ In addition to these mononuclear complexes, fac-[Re­(CO)_3_]^+^ dimers described Ramakrishna et al.^58^ or Low et al.^59^ For example, phenanthroline-based derivatives have shown significant activity against cancer cells, including the work by Enslin et al.^60^

**Figure 4:**
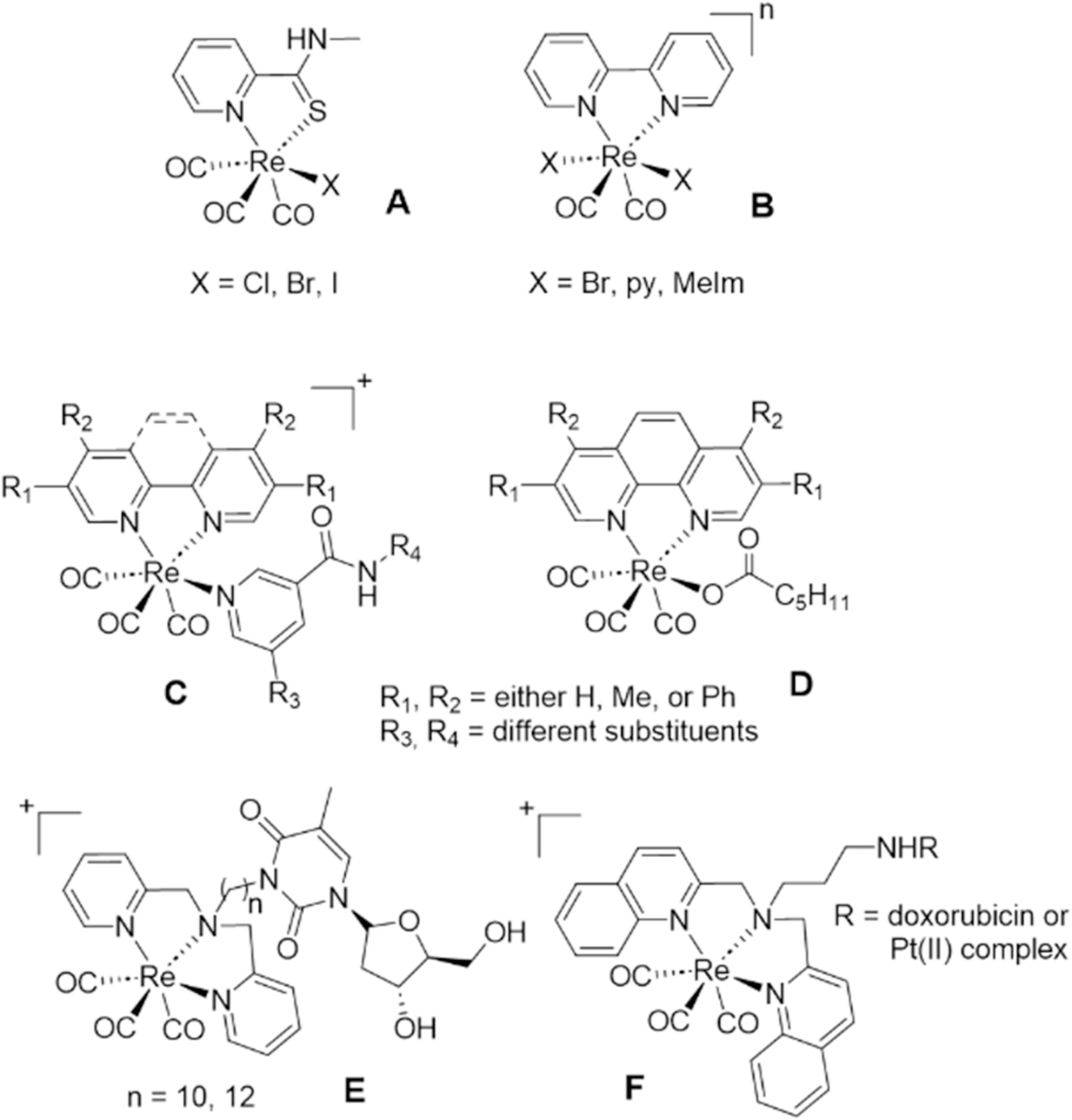
Representative structures of rhenium­(I) tricarbonyl complexes employed in medicinal and bioinorganic chemistry. Complexes (A–F) illustrate the structural diversity accessible through ligand modification, including mono- and polypyridyl systems, Schiff-base derivatives, polymeric architectures, and drug-conjugated compounds. Variations in the coordination environment and ligand framework enable tuning of photophysical, redox, and biological properties for imaging, therapeutic, and catalytic applications.

Linking ligand characteristics—such as size, denticity, π-conjugation, and functionalization—strongly affects the cytotoxic behavior of the represented motifs based on Re­(I) tricarbonyl complexes. The above-mentioned features of the ligand can be commented on in the following manner when attempting to link them to the cytotoxic effects of Re­(I) tricarbonyl complexes. Considering the size of the ligand, we can track how it influences the overall steric environment around the Re metal center. When the Re center is in the field of larger ligands, this is reflected in the creation of steric hindrance, which is directly linked to a complex’s ability to interact with biological targets such as proteins, thereby impacting the cytotoxic response. Ligands with more extended π-conjugation can affect the electronic properties of the complex, such as its redox potential. However, in our case the balance between conjugation and steric bulk can provide useful properties, such as improved cellular uptake or selectivity.

To quantitatively explore descriptor–cytotoxicity relationships, Figure 5 presents raincloud plots of the top 10 molecular descriptors selected according to their importance scores, comparing the distributions between noncytotoxic (cluster 0) and cytotoxic (cluster 1) Re­(I) tricarbonyl complexes.

**Figure 5:**
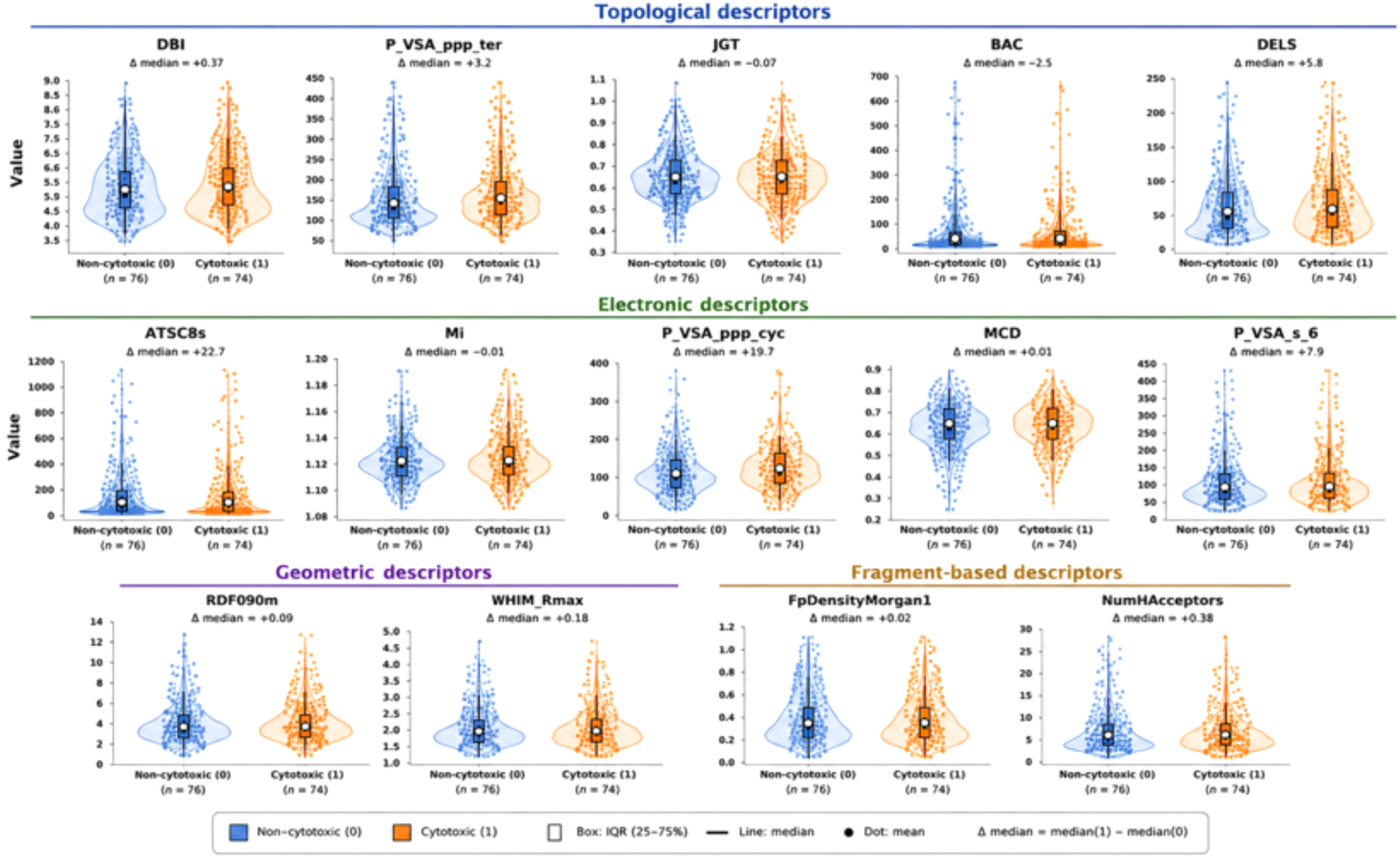
Raincloud plot representation of the selected molecular descriptors showing their distributions across cytotoxic and noncytotoxic Re­(I) tricarbonyl complexes. The plots illustrate differences in central tendency, dispersion, and population overlap, providing insight into descriptor–cytotoxicity relationships.

The molecular descriptors were grouped into topological, electronic, geometric, and fragment-based categories (Table 1) to facilitate interpretation of the structural and physicochemical determinants associated with cytotoxic behavior. These descriptor classes capture complementary aspects of molecular organization that may influence biological activity through distinct mechanisms.

**Table 1:** Representation of the Descriptors Contributing to the Discrimination between Cytotoxic and Non-Cytotoxic Rhenium­(I) Tricarbonyl Complexes

descriptor type	meaning	implication for toxicity
DBI (Distance-based topological index)	captures molecular branching and size	higher value more cytoxic class may suggest more extended/bulky structures
P_VSA_ppp_ter/cyc/con/L	volume-based surface area (polarizable fragments)	differences may reflect varied exposure of polar or reactive groups
ATSC 1s, ATSC8s	autocorrelation descriptors weighted by sigma electrons	indicates spatial distribution of electron properties
SpAD_EA(dm)/SpMAD_EA (dm)	eigenvalue-weighted edge adjacency indices	related to overall connectivity and molecular flexibility
nBM (number of bonds in molecule)	simple complexity measure	higher values could imply denser frameworks linked to bioactivity
TIE (Topological Information Content)	diversity of atom types	higher values in toxic might reflect complex or diverse functionalization
SM1_Dz(p), SM1_Dz(i)	electrotopological state descriptors	encode atomic and electronic environments relevant for binding or disruption
EE_B(s), Eta_B	electrophilic behavior	more reactive compounds may increase off-target or membrane-disruptive toxicity
C%	carbon percentage	could reflect lipophilicity or scaffold type
SpMax6_Bh(s), SpMax7_Bh(s)	maximal electrotopological values	strong peaks could be linked to specific reactive sites

Raincloud plots combine violin distributions (density estimation), individual observations (jittered data points), and summary statistics (median and interquartile range), enabling simultaneous visualization of distribution shape, central tendency, variability, and outliers.

From a mechanistic perspective, differences in descriptor distributions is reflecting on molecular properties that influence cellular response and therefore contribute to cytotoxicity. Topological descriptors describe molecular connectivity and complexity, which can affect transport and target accessibility. Electronic descriptors relate to charge distribution, polarity, and reactivity, influencing interactions with biomolecules and potential induction of oxidative or chemical stress. Geometric descriptors characterize molecular size, shape, and steric organization, affecting membrane crossing and binding complementarity. Fragment-based descriptors capture the occurrence of specific structural motifs that may promote or reduce biological activity. Together, these factors influence processes such as cellular uptake, membrane permeability, molecular recognition, intracellular accumulation, and target interaction, which collectively contribute to the observed cytotoxicity patterns.

Figure 5 illustrates the distribution of the top-ranked descriptors across clusters, showing largely overlapping ranges with modest shifts in central tendency. Table 2 also present. Notably, descriptors such as DBI, ATSC8s, P_VSA_ppp_ter, and Eig03_AEA (dm) show clear separation in their value distributions, suggesting that cytotoxic compounds tend to exhibit more complex topologies, greater polarizability, and enhanced electron delocalization. Similarly, Eta_B, SpMax6_Bh(s), and SpMAD_EA­(dm) indicate higher electrophilic potential and branching in the cytotoxic class, implying a greater likelihood of biochemical reactivity or cellular interaction. These features highlight that cytotoxic rhenium complexes generally possess larger, more branched, and electronically rich frameworks—making these descriptors effective markers and structural fingerprints for predictive modeling and rational compound design.

**Table 2:** Discriminator Descriptors List Based on Box-Plot Analysis^a^

descriptor	type	interpretation of discriminatory pattern recognition
DBI	topological Index	higher in cytoxic → larger, complex molecules.
ATSC8s	autocorrelation (sigma)	shifted higher in cytoxic → extended polarizability.
P_VSA_ppp_ter	polar surface area (terminal)	higher → more exposed polar groups.
Eig03_AEA(dm)	edge adjacency/distance	elevated in cytoxic → more delocalized electrons.
Eta_B	electrophilic reactivity	higher in cytoxic → greater redox potential.
SpMax6_Bh(s)	branching/hydrophobicity	clear separation → toxic molecules more branched.
SM1_Dz(i)	electrotopological index	cytoxic class shows distinct profile.
P_VSA_ppp_con	surface area (conjugated)	higher in cytoxic → reflects extended conjugation.
SpMAD_EA(dm)	topological reactivity	cytoxic values well-separated → increased activity.
nBM	number of bonds	more bonds in cytoxic compounds → larger scaffolds.

a All descriptors, are structure-derived topochemical or physicochemical indices calculated uniformly for all compounds using alvaDesc,^38^ without the use of any experimental or quantum-chemical input data.

Box plot analysis of the top 40 descriptors (with all box plots presented in the SI) reveals structure–cytotoxicity relationships in rhenium­(I) tricarbonyl complexes. In contrast, noncytotoxic complexes generally feature more compact and inert molecular scaffolds, as reflected by lower values across several descriptors.

Notably, this structure-based discrimination was achieved solely through the application of the K-means clustering algorithm, with the entire interpretation relying exclusively on descriptor patterns—without the need for labeled groups during training. We chose to utilize and explore the advantages of K-means over some of the existing supervised learning algorithms due to their elegance and simplicity in data mining, particularly when working in small data sets regimes. This is especially relevant in the field of metal-based drug development, where the available biological data is often limited. The main message conveyed from the analysis of molecular descriptors, as summarized in Table 2, reveals distinct patterns that differentiate cytotoxic from noncytotoxic Re­(I) tricarbonyl complexes. Key topological, electronic, and geometric features emerge as critical factors influencing the cytotoxicity response.

## Conclusions

This study shows that unsupervised machine learning—specifically K-means clustering—can be a useful and practical way to explore structure–cytotoxicity relationships in rhenium­(I) tricarbonyl complexes, especially when working with limited and heterogeneous data. Instead of forming clearly separated groups, the compounds are distributed across a continuous chemical space, where cytotoxicity emerges from a combination of structural and electronic features rather than a single defining property.

We observe clear trends: certain regions of chemical space are enriched in cytotoxic or noncytotoxic compounds, indicating that meaningful structure–activity relationships can still be identified. The descriptors that drive this separation point to factors such as molecular size, electronic distribution, and surface properties, which are likely linked to how these complexes interact with biological systems.

These results highlight an important point for metal-based drug discovery: when consistent biological data are not available, unsupervised approaches are not just an alternative, but a practical and informative strategy. Rather than focusing on prediction alone, they allow us to understand how chemical structure relates to biological behavior in a more flexible and interpretable way.

Overall, this approach provides a straightforward tool for early stage screening and for guiding the design of new metallodrugs. Expanding this framework to larger data sets and combining it with more advanced or hybrid methods could further improve both predictive power and mechanistic insight.

## Supplementary Material



## Data Availability

Data set are included in the linked repository https://zenodo.org/records/17940325

## Notes

Experimental section; IR spectra; UV–vis spectra; ^1^H NMR spectra; HeLa cell viability data; descriptor box plots for cytotoxic and noncytotoxic Re­(I) tricarbonyl complexes (PDF)
