# Supplementary material for: Unlabeled but Not Unseen: Cytotoxicity Classification of Re(I) Tricarbonyl Complexes via K‑Means Clustering
Source: ACS Omega. 2026 Jun 11;11(24):35208–17. doi: 10.1021/acsomega.5c13156 (PMC13295016; doi:10.1021/acsomega.5c13156)
Supplement: Supplementary file 1 [file ao5c13156_si_001.pdf]

SUPPORTING INFORMATIONS FOR

Unlabeled but Not Unseen: Cytotoxicity Classification of Re(I) Tricarbonyl Complexes via K-Means Clustering

Miroslava Nedyalkova,[1,2,3,4#*] Gozde Demirci,[1#] Youri Cortat,[1] Lilai Abraha,[1] Aurelien Crochet,[1] Marco Lattuada,[1] and Fabio Zobi[1*]

[1]Department of Chemistry, University of Fribourg, Chemin Du Musée 9, 1700 Fribourg, Switzerland

[2] Swiss National Center for Competence in Research (NCCR) Bio-inspired Materials, University of Fribourg, Fribourg, Switzerland

[3] Faculty of Chemistry and Pharmacy, Sofia University "St. Kliment Ohridski", 1 James Bourchier Blvd., 1164 Sofia, Bulgaria.

[4] Research and Development and Innovation Consortium (Sofia Tech Park), 111 Tsarigradsko Shosse Blvd., 1784 Sofia, Bulgaria

[#] These authors equally contributed.

Table of contents

Experimental section – page 2-4

IR spectra (solid state) of complexes – Figure S1 – page 4

UV-Vis spectra of complexes – Figure S2 – page 5

^{1}H-NMR spectra of complexes – Figures S3-S9 – page 6-8

HeLa cells viability data treated with complexes **1a**-**6b** – Figure S10 – page 8

Experimental section

Reagents and instruments. All chemicals were used in high purity. For bioactivity experiments, all materials were used properly. IR measurements were performed with a Bruker TENSOR II with the following parameters: 16 scans for the background and 32 scans for the sample with a resolution of 4 cm^{-1} in the 4000 to 600 cm^{-1} region. UV-Vis spectra of the complexes were measured on a Jasco V730 spectrophotometer. NMR measurements were performed with a Bruker Advance III 400 MHz instrument. Cytotoxicity test was validated by using Tecan-Infinity M Nano with iControl program. HeLa cells were kindly gifted from Prof. David Hoogewijs, Department of Medicine, University of Fribourg, Switzerland. Single crystal diffraction data were obtained using an Oxford Cryosystems cryostat coupled to a Stoe STADIVARI diffractometer (CuKα1 (λ = 1.5406 Å)). Structures were solved using Intrinsic Phasing in the ShelXT structure solution and refined using Least Squares minimization in the ShelXL refinement software.

General preparation of *fac*-[Re(CO)$_3$(NN)L]CF$_3$SO$_3$ complexes 3b, 4b, 6b.

To a solution of the appropriate *fac*-[Re(CO)$_3$NNBr] complex (1.0 equiv.) in methanol under argon, AgCF$_3$SO$_3$ was added and refluxed for 8-12 h, then the solution was filtered to remove AgBr from the reaction mixture. The next step was performed by adding methylimidazole to a solution of *fac*-[Re(CO)$_3$NN#(CF$_3$SO$_3$)] in MeOH, then the reaction mixture was refluxed under argon for 10 h, and finally filtered. The filtrate was evaporated and the yellow residue was purified over an alumina column using ethyl acetate: methanol (100:2-10%) as the mobile phase. The purity of the complexes was evaluated by ^{1}H-NMR. All complexes showed a purity of at least > 95% by this technique.

General preparation of *fac*-[Re(CO)$_3$(NN)Ben] complexes 1c, 3c, 4c.

The *fac*-[Re(CO)$_3$(NN)Ben] carboxylato complexes were prepared according to the following procedure. *fac*-[Re(CO)$_3$(NN)Br] (100 mg, 0.2 mmol) and benzoic acid (25mg, 0.2 mmol) were dissolved in anhydrous degassed tetrahydrofuran (30 mL). The reaction mixture was stirred for 5 min, then

trimethylamine (33 µL, 24 mg, 0.24 mmol) and $AgCF_3SO_3$ (50 mg, 0.2 mmol) were added, and the mixture stirred at 70 °C for 18 h. The mixture was filtered, solvent evaporated to dryness and the residue purified by column chromatography on deactivated alumina with DCM as the eluent. Analytically pure products were obtained after recrystallization.

***fac*-[Re(CO)$_3$(bpy)Ben] (1c):** Yellow powder, yield 47%. IR (solid, ν_{CO}, cm^{-1}): 2012.12, 1866.45. UV-Vis (MeOH, λ_{max} [nm]): 277, 261. ^{1}H NMR (400 MHz, CD_3CN): δ = 9.08 - 9.14 (m, 2 H), 8.42 (d, *J*=8.2 Hz, 2 H), 8.20 (td, *J*=7.9, 1.6 Hz, 2 H), 7.62 (ddd, *J*=7.7, 5.5, 1.3 Hz, 2 H), 7.35 - 7.41 (m, 2 H), 7.24 (d, *J*=7.5 Hz, 1 H), 7.09 - 7.16 ppm (m, 2 H). Crystallization of the complex was performed by layering heptane on a DCM solution of the complex. CCDC #: 2466407

***fac*-[Re(CO)$_3$(tmetphen)MeIm](CF$_3$SO$_3$) (3b):** Yellow powder, yield 36.6%. IR (solid, ν_{CO}, cm^{-1}): 2026.27, 1904.92. UV-Vis (MeOH, λ_{max} [nm]): 280, 250. ^{1}H NMR (400 MHz, CD_3CN): δ = 9.23 (s, 2 H), 8.31 (d, *J*=1.6 Hz, 2 H), 7.29 (s, 1 H), 6.73 (d, *J*=1.3 Hz, 1 H), 6.47 (d, *J*=1.3 Hz, 1 H), 3.39 (d, *J*=1.1 Hz, 3 H), 2.82 (s, 6 H), 2.64 ppm (s, 6 H). ^{13}C-NMR (400 MHz, d-DMSO): δ = 154.56 (1 C), 148.16 (2 C), 144.21 (1 C), 140.84 (1 C), 135.64 (2 C), 128.85 (2 C), 128.27 (2 C), 124.19 (2 C), 122.76 (2 C), 33.99 (1 C), 17.28 (2 C), 15.01 (2 C) ppm. ESI-MS (MeOH): *m/z*, 589.0 $[M]^+$, measured; calculated for $[C_{23}H_{22}N_4O_3Re]^+$: 588.66. Crystallization of the complex was performed by layering heptane on a DCM solution of the complex. CCDC #: 2466410

***fac*-[Re(CO)$_3$(tmetphen)Ben] (3c):** Yellow powder, yield 3%. ^{1}H NMR (400 MHz, CD_3CN): δ = 9.19 (s, 2 H), 8.29 (s, 2 H), 7.13 - 7.18 (m, 3 H), 6.96 - 7.02 (m, 2 H), 2.80 (s, 6 H), 2.60 ppm (s, 6 H). All sample was used for biological experiments. Crystallization of the complex was performed by slow evaporation of a ACN solution of the complex. CCDC #: 2466411

***fac*-[Re(CO)$_3$(dimetphen)MeIm](CF$_3$SO$_3$) (4b):** Yellow powder, yield 56.4%. IR (solid, ν_{CO}, cm^{-1}): 2046.37, 1921.10, 1890.58. UV-Vis (MeOH, λ_{max} [nm]): 369, 284, 252.5. ^{1}H NMR (400 MHz, CD_3CN): δ = 8.59 (d, *J*=8.4 Hz, 2 H), 8.01 (s, 2 H), 7.97 (d, *J*=8.4 Hz, 2 H), 7.10 (s, 1 H), 6.70 (s, 1 H), 6.21 (s, 1 H), 3.35 (s, 3 H), 3.28 ppm (s, 6 H). ^{13}C-NMR (400 MHz, d-DMSO): δ = 195.58 (1 C), 164.41 (2 C), 146.33 (2 C), 141.24 (1 C), 140.08 (2 C), 128.76 (2 C), 127.40 (2 C), 126.85 (2 C), 122.83 (1 C), 33.90 (1 C), 30.54 (2 C) ppm. ESI-MS (MeOH): *m/z*, 561.0 $[M]^+$, measured; calculated for $[C_{21}H_{18}N_4O_3Re]^+$: 560.61. Crystallization of the complex was performed by layering heptane on a DCM solution of the complex. CCDC #: 2466412

***fac*-[Re(CO)$_3$(dimetphen)Ben] (4c):** Yellow powder, yield 10%. IR (solid, ν_{CO}, cm^{-1}): 2012.12, 1866.45. UV-Vis (MeOH, λ_{max} [nm]): 277, 261. ^{1}H NMR (400 MHz, CD_3CN): δ = 8.52 (d, J=8.4 Hz, 2 H), 7.97 (s, 2 H), 7.85 (d, J=8.3 Hz, 2 H), 7.12 - 7.17 (m, 1 H), 6.93 - 6.99 (m, 2 H), 6.87 - 6.90 (m, 2 H), 3.32 ppm (s, 6

H). Crystallization of the complex was performed by layering heptane on a DCM solution of the complex. CCDC #: 2466413

***fac*-[Re(CO)$_3$(bathocup)Br] (6a):** Yellow powder, yield 87%. IR (solid, ν_{CO}, cm^{-1}): 2011.54, 1880.33, 1862.70. UV-Vis (MeOH, λ_{max} [nm]): 292. ^{1}H NMR (400 MHz, CD_3CN): δ = 7.95 (s, 2 H), 7.89 (s, 2 H), 7.62 (s, 10 H), 3.34 ppm (s, 6 H). ^{13}C-NMR (400 MHz, $CDCl_3$): δ = 162.97 (2 C), 150.83 (2 C), 149.04 (2 C), 135.93 (2 C), 129.46 (6 C), 129.02 (4 C), 127.29 (2 C), 126.62 (2 C), 124.36 (2 C), 31.47 (2 C).

***fac*-[Re(CO)$_3$(bathocup)MeIm](CF$_3$SO$_3$) (6b):** Yellow powder, yield 97.5%. IR (solid, ν_{CO}, cm^{-1}): 2021.97, 1896.36. UV-Vis (MeOH, λ_{max} [nm]): 295, 260. ^{1}H NMR (400 MHz, CD$_3$CN): δ = 7.96 (s, 2 H), 7.93 (s, 2 H), 7.63 (s, 10 H), 7.23 (s, 1 H), 6.78 (s, 1 H), 6.35 (s, 1 H), 3.43 (s, 3 H), 3.30 ppm (s, 6 H). ^{13}C-NMR (400 MHz, d-DMSO): δ = 195.69 (1 C), 163.87 (2 C), 150.82 (2 C), 147.46 (2 C), 141.48 (1 C), 135.19 (2 C), 129.67 (6 C), 129.08 (4 C), 127.58 (2 C), 126.53 (2 C), 124.65 (1 C), 123.03 (2 C), 34.01 (1 C), 30.51 (2 C). ESI-MS (MeOH): *m/z*, 713.0 [M]$^+$, measured; calculated for [C$_{33}$H$_{26}$N$_4$O$_3$Re]$^+$: 712.8.

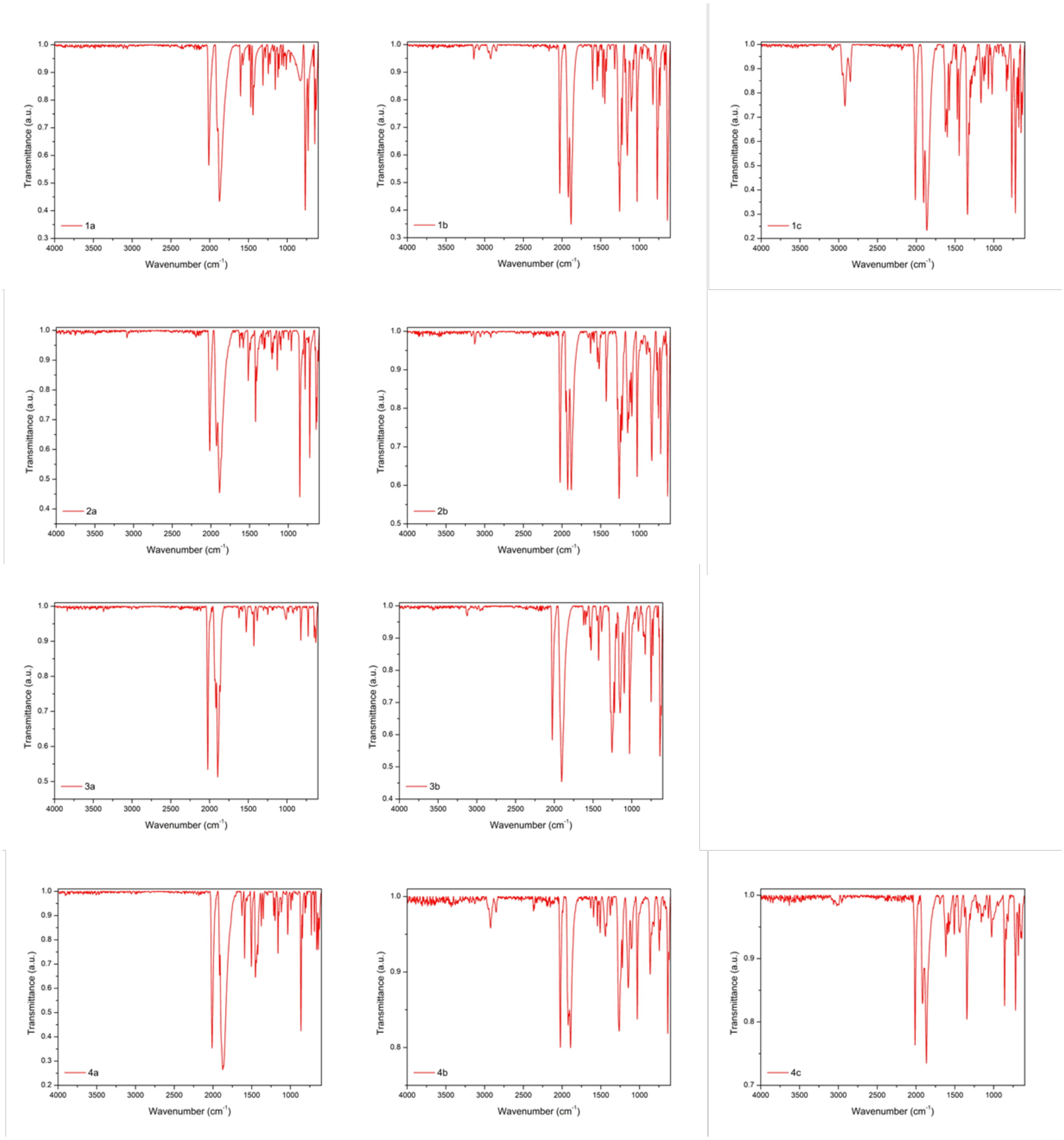

Figure S1: IR spectrum of **1a, 1b, 1c, 2a, 2b, 3a, 3b, 4a, 4b,** and **4c**.

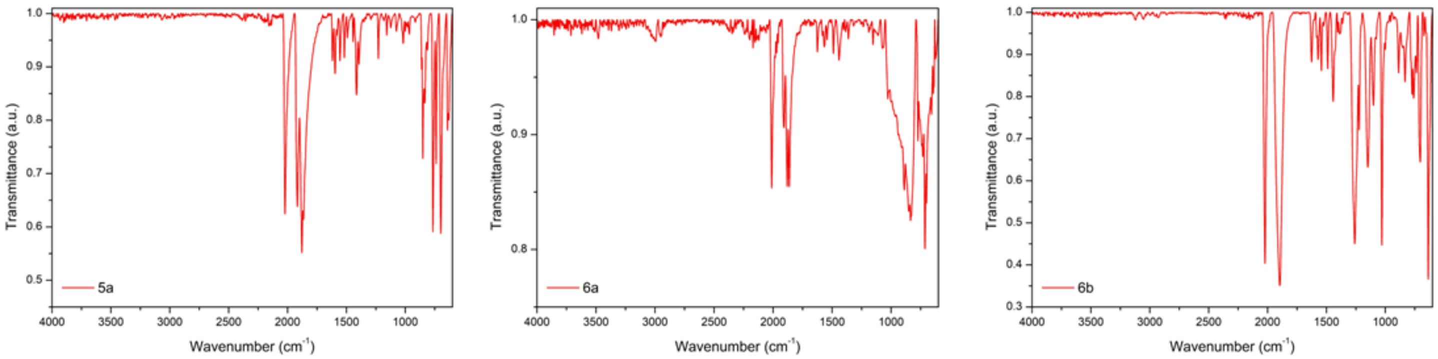

Figure S1 (continued): IR spectrum of **5a, 6a** and **6b**.

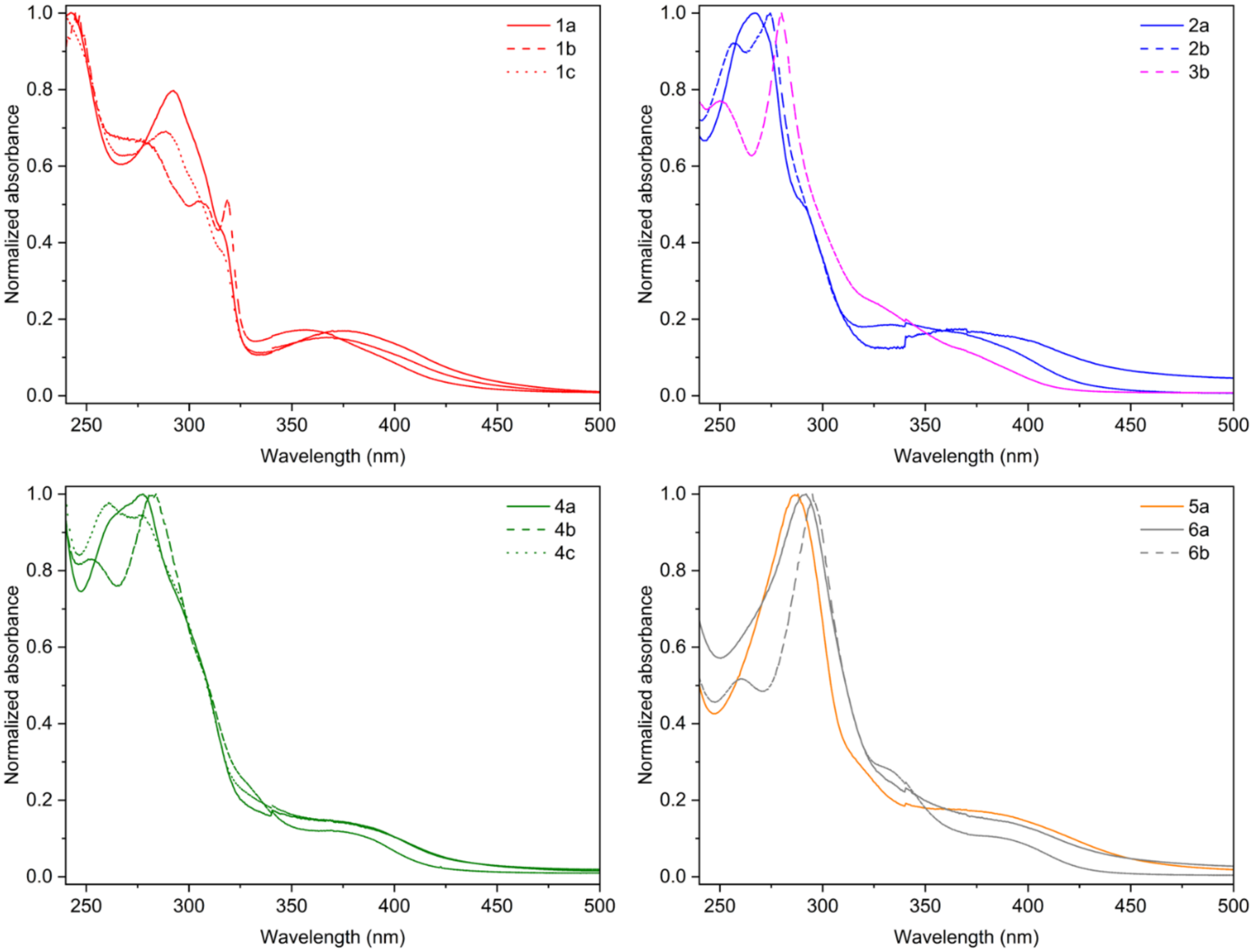

Figure S2: UV spectrum of complexes.

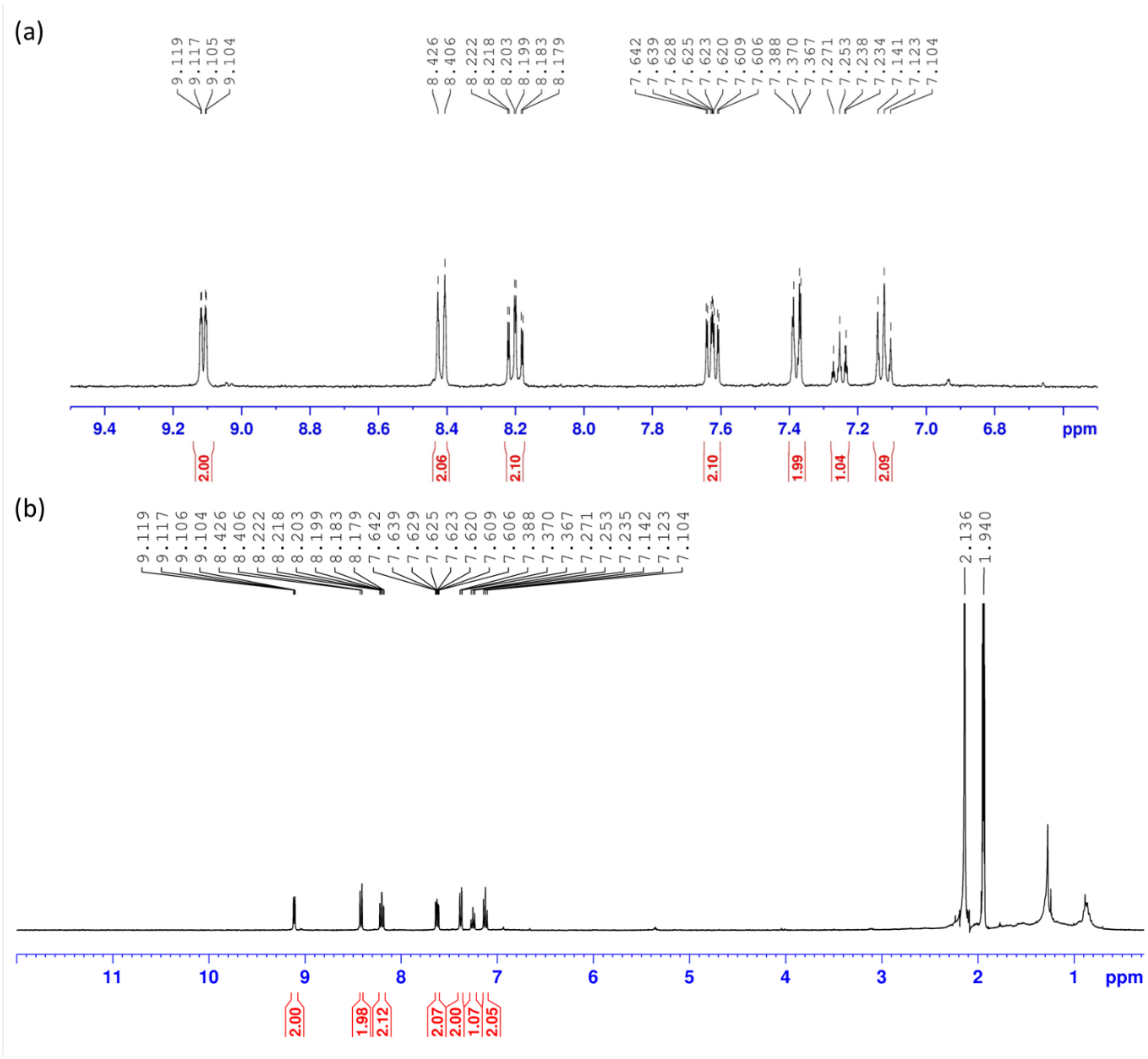

Figure S3: Selected region (a) and full (b) ^{1}H-NMR spectrum of **1c** complexes in d-ACN.

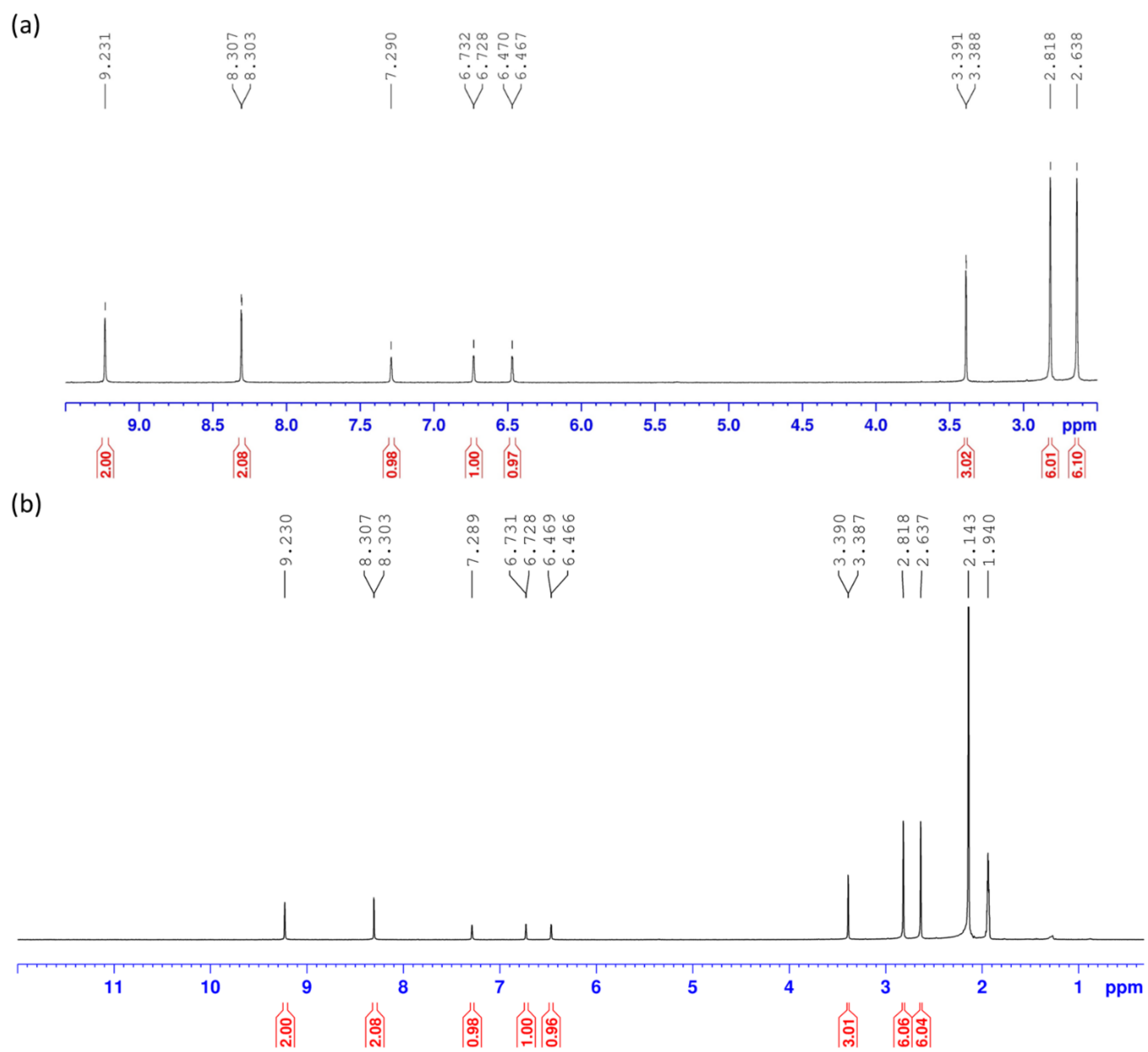

Figure S4: Selected region (a) and full (b) ^{1}H-NMR spectrum of **3b** complexes in d-ACN.

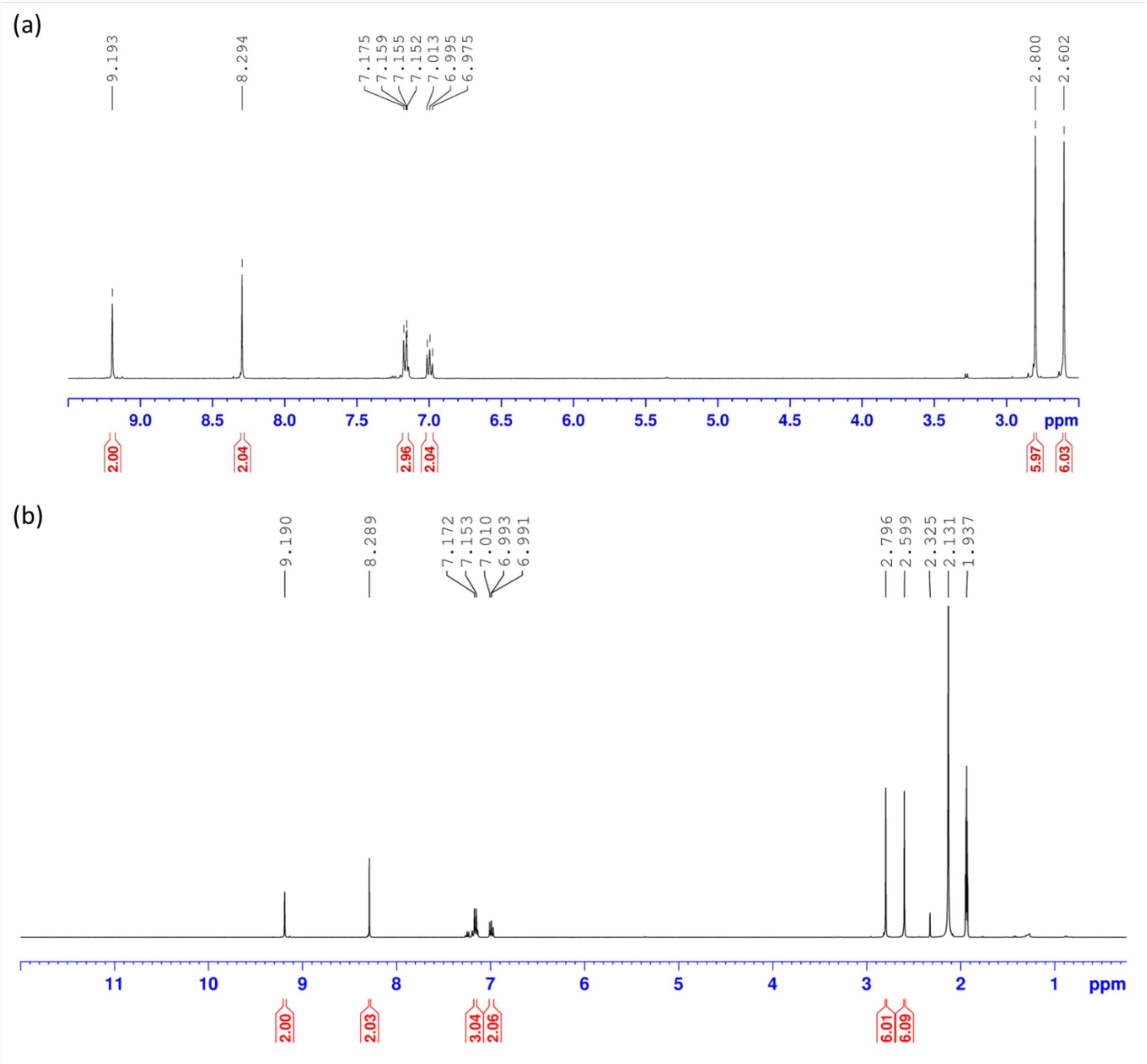

Figure S5: Selected region (a) and full (b) ^{1}H-NMR spectrum of **3c** complexes in d-ACN.

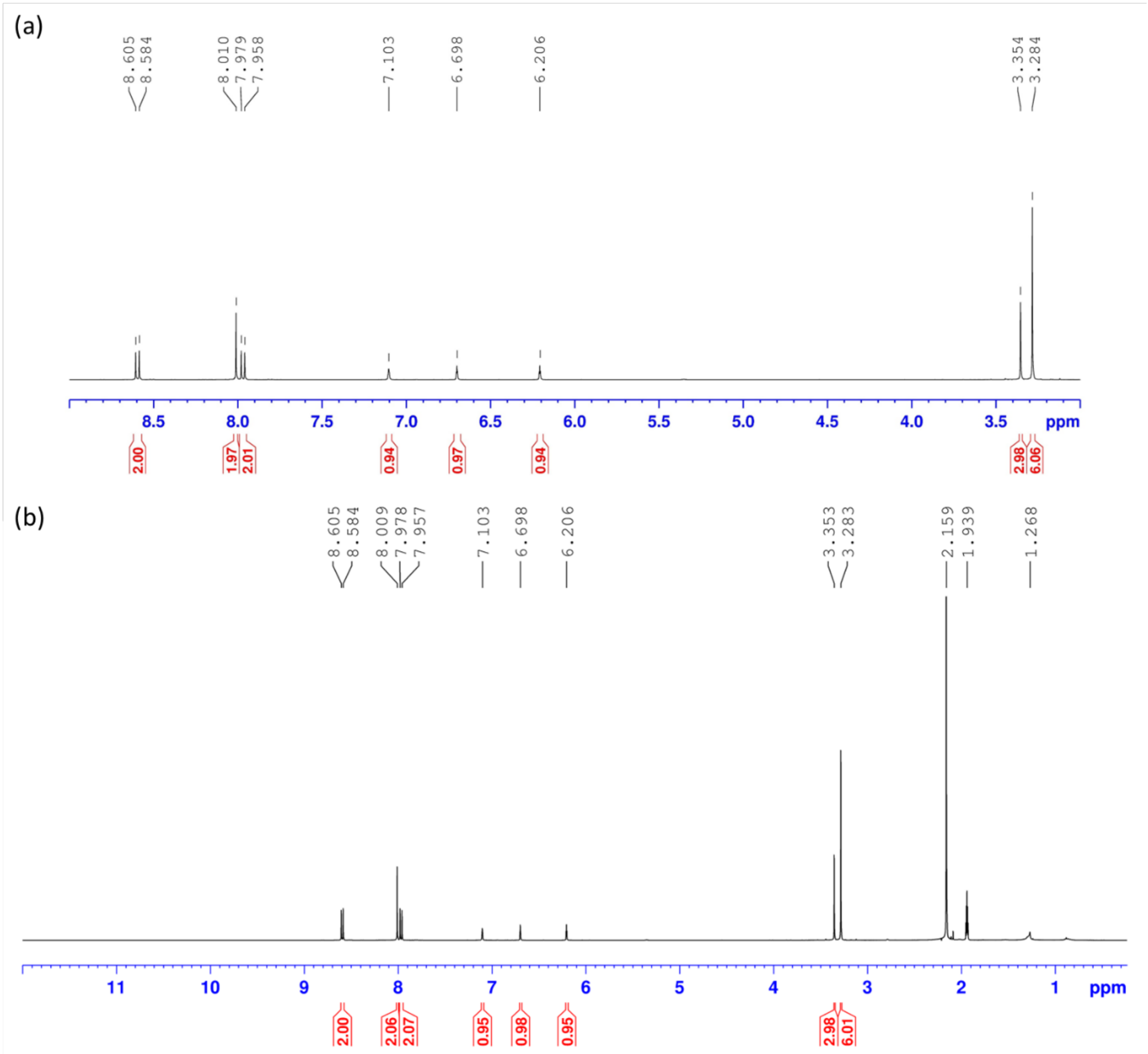

Figure S6: Selected region (a) and full (b) ^{1}H-NMR spectrum of **4b** complexes in d-ACN.

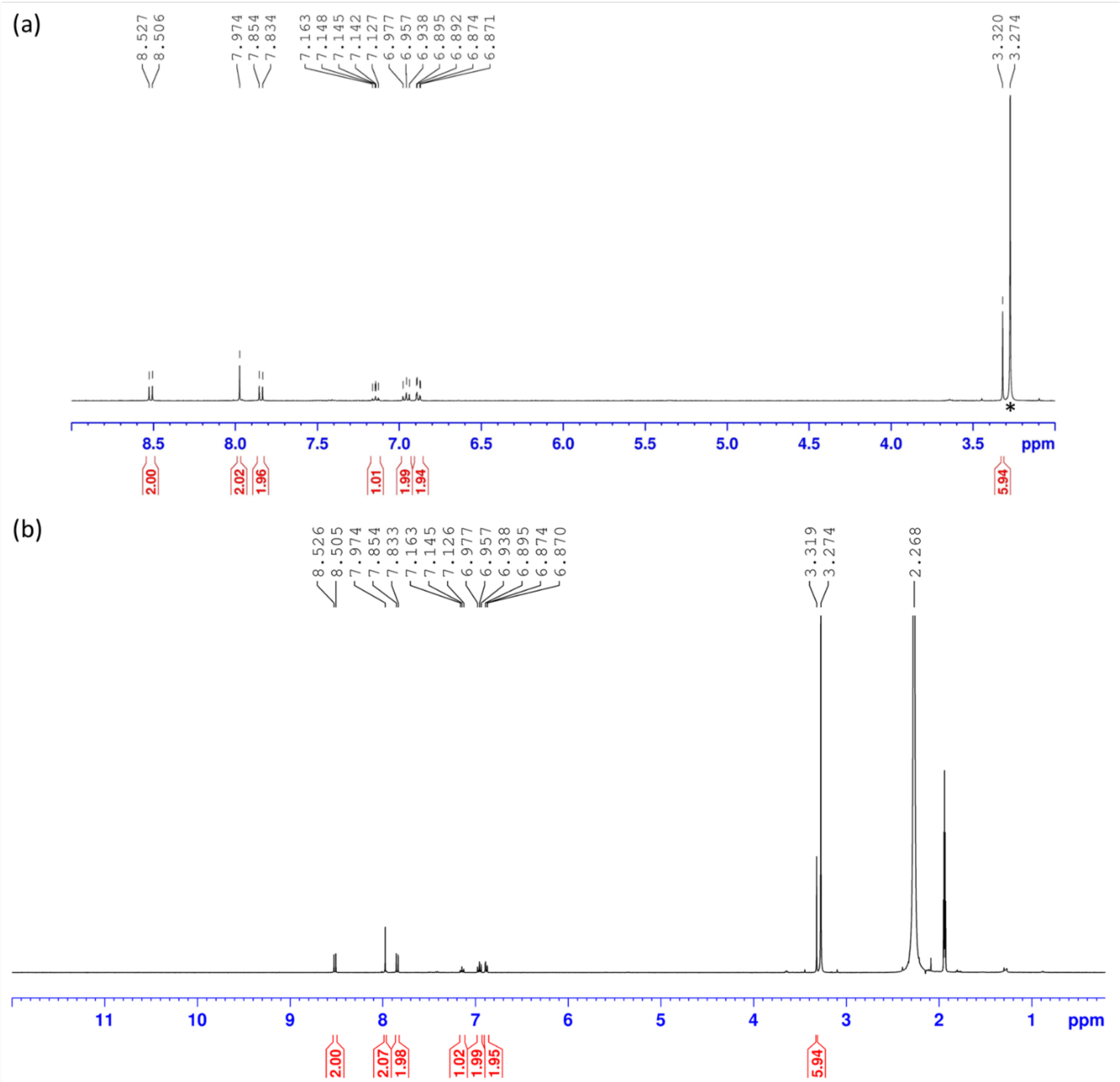

Figure S7: Selected region (a) and full (b) ^{1}H-NMR spectrum of **4c** complexes in d-ACN. (*MeOH)

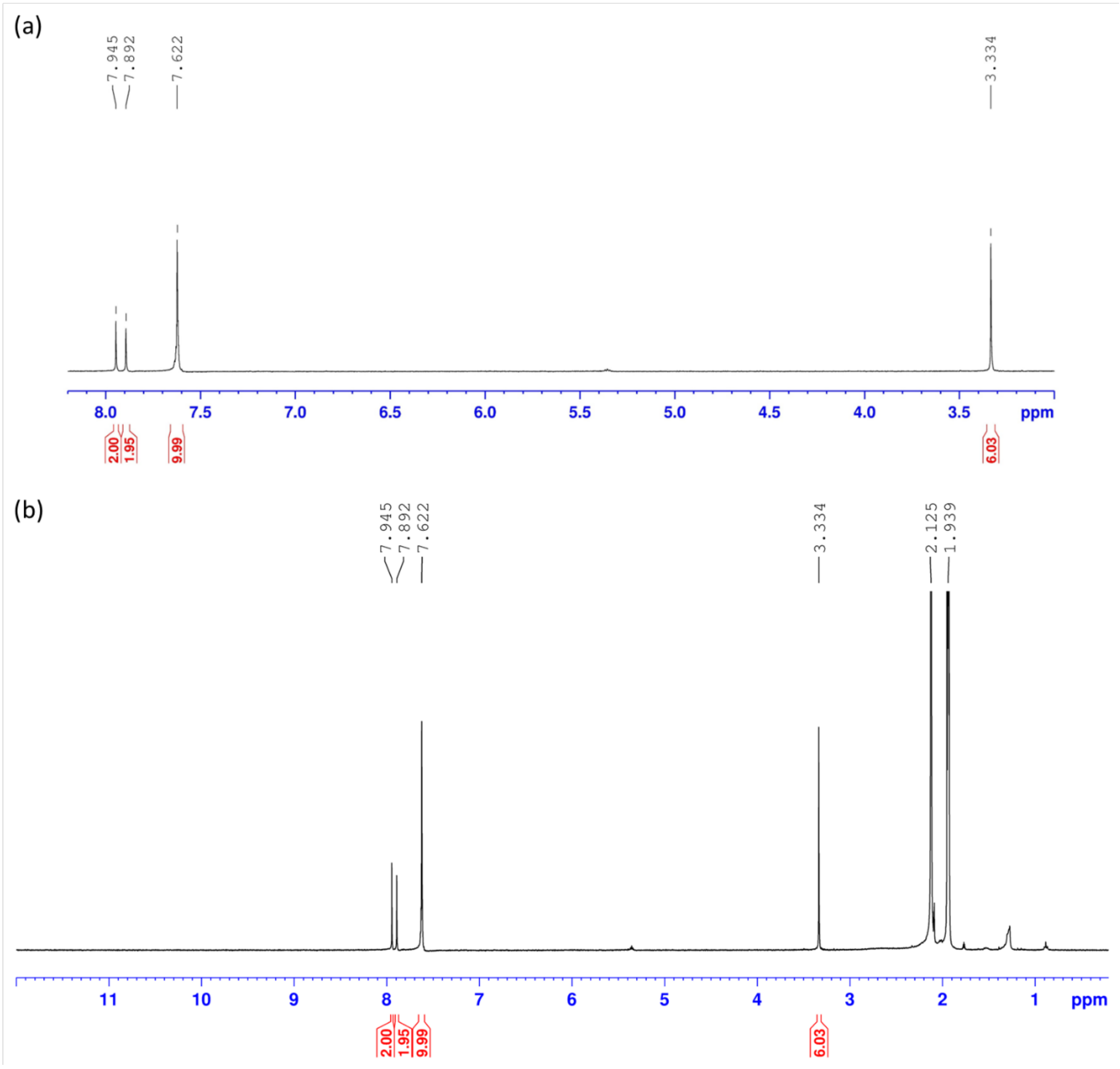

Figure S8: Selected region (a) and full (b) ^{1}H-NMR spectrum of **6a** complexes in d-ACN.

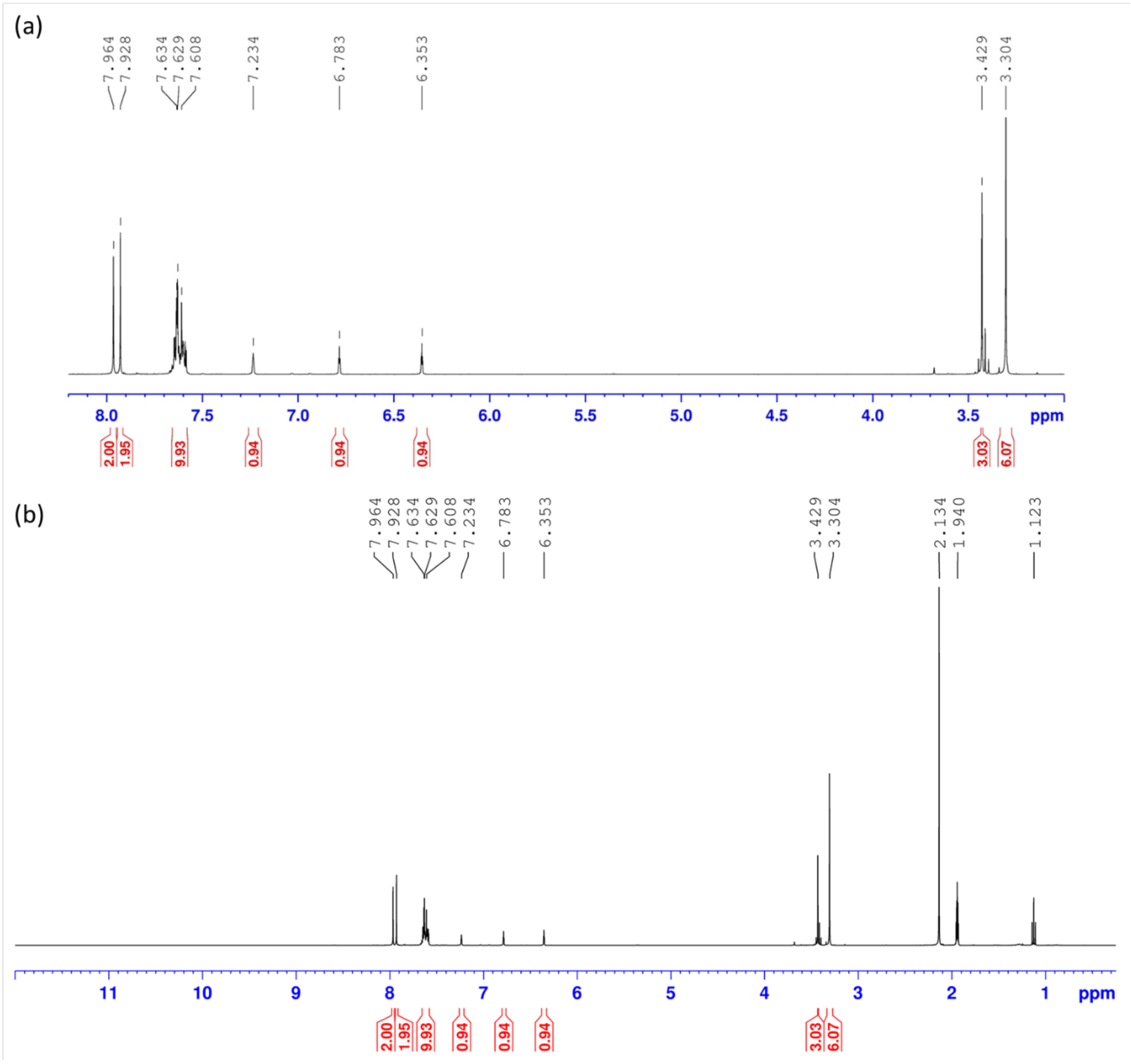

Figure S9: Selected region (a) and full (b) ^{1}H-NMR spectrum of **6b** complexes in d-ACN.

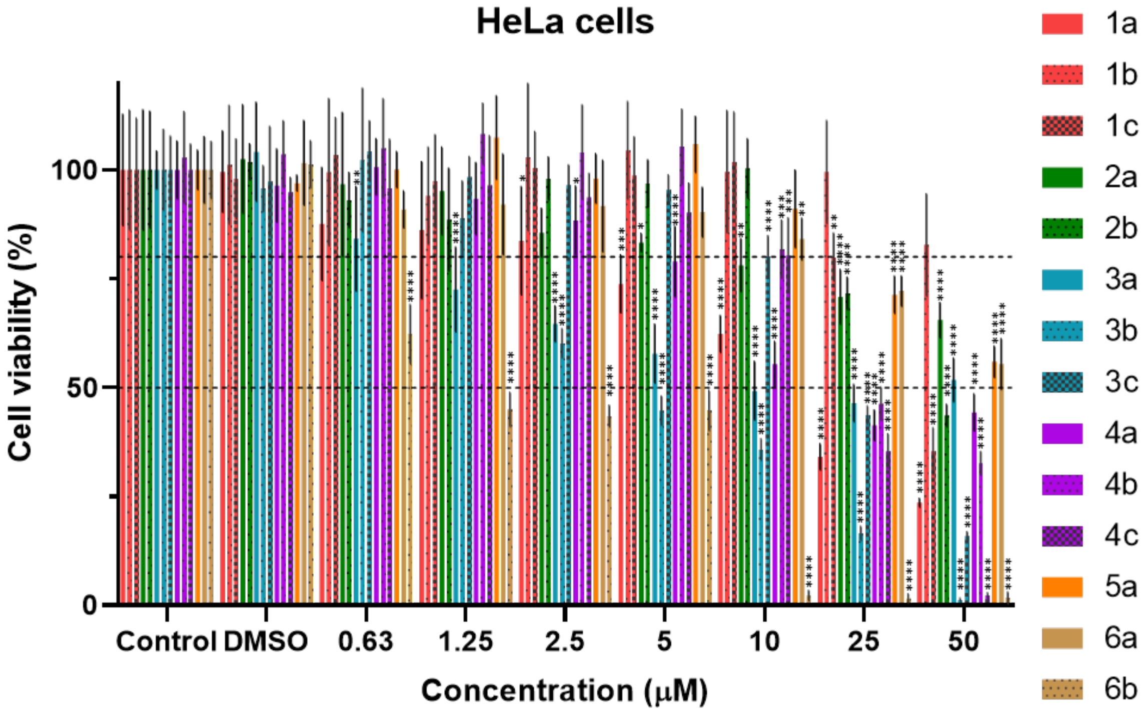

Figure S10: HeLa cells viability data treated with complexes **1a**-**6b**

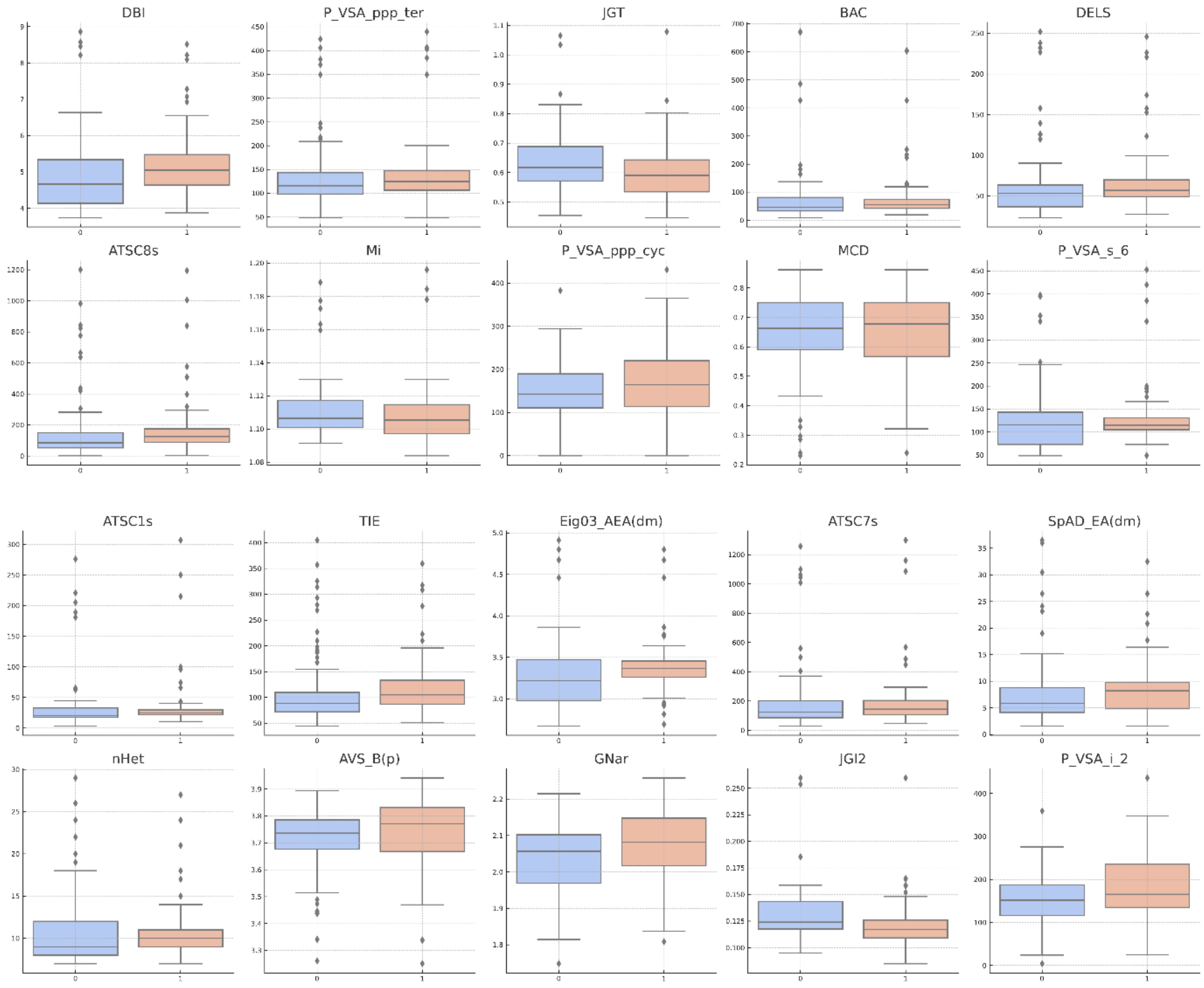

Figure S11: Box plots of the forty descriptors contributing to the discrimination between cytotoxic (1-blue) and non-cytotoxic (0-orange) Re(I) tricarbonyl complexes.

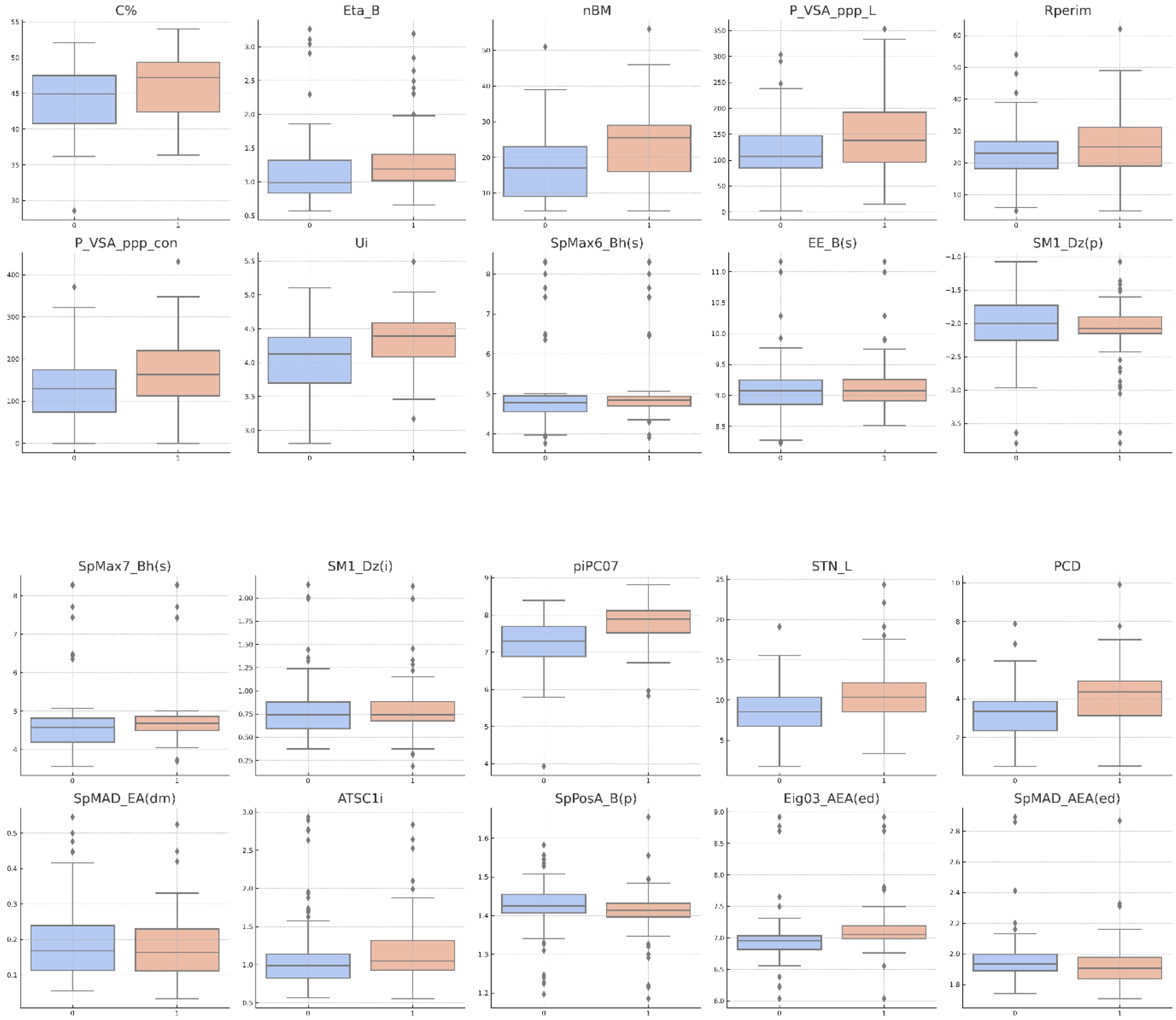

Figure S11 (continued): Box plots of the forty descriptors contributing to the discrimination between cytotoxic (1-blue) and non-cytotoxic (0-orange) Re(I) tricarbonyl complexes.
